# The Essays, Cases, Intelligence, &c.: S—Z

**Published:** 1820

**Authors:** 


SAAMAR, Dr. case in which the deleterious effects of opium were remedied by the excitement of pain, iii. 574.

SABATIER, Professor, cases of hydrophobia, with remarks, xvii. 218, 344; observations on fracture of the sternum, xviii. 20.

SACCO, Dr. observations on the practice of vaccination in Lombardy, vii. 109; review of his work on cow-pox, 169.

SAFNER, Mr. observations on the use of turpentine as a vermifuge, xxv. 168.

SAGE, Mr. observations on the narcotic effluvia of plants, xviii. 288.

SAGITTARIA, botanical description and medical qualities of the, xvii. 466.

SAINSBURY, Dr. on the influenza of 1803, x. 115.

SAL-AERATUS, process for its preparation, i. 295.—*See* POTASH.

SAL-AMMONIAC, observations on its use in various surgical cases, xiii. 497.

SALAMANDER, natural history of the, v. 177.

SALDER, Dr. observations on yellow fever,

SALISBURY, Mr. observations on the prevalent diseases of the year 1809, xxiv. 433.

SALIVATION. — *See* PTYALISM, *and* MERCURY.

SALMON, Mr. Thomas, of Salisbury, his method of clearing apartments from noxious air, viii. 575.

————, Mr. of Hanway, description of his ventilator for hospitals, xi. 101.

SALSOLA, botanical description and medical qualities of, xii. 229.

SALTS, account of a new species of, iii. 82; good effects of the alkaline in counteracting the agency of corrosive sublimate, 535; discovery of a new species, the pneumalkli, iv. 446; the hydro-sulphate of soda, 469; account of some new experiments on the mercurial, ix. 59.

SALTER, Mr. T. history of a disease of the uterus, xxx. 337; case of disease of the female breast cured by the use of iron and kali purum, xxxiii. 115; case of disease of the brain, xxxiv. 519.

SAMBUCUS, botanical description and medical qualities of, xxii. 558.

SANCHO, Mr. report of cow-pox institution, xii. 90.

SANDERS, Mr. J. Cunningham, observations on inflammation of the iris, xvi. 55; review of his treatise on diseases of the eye, xxvii. 228, 335; remarks on his work on the ear, xviii. 5.

SANDFORD, Mr. account of the remarkable structure of the organs of generation in a calf, ii. 305; observations on the influenza of 1803, x. 223.

SANGUINARIA, Canadense, botanical description and medical qualities of, xxv. 166.

SANTRY, Mr. observations on preternatural labour, xxx. 341; observations on phlegmasia dolens, xxxii. 524.

SAPON, M. historical view of the prevalence of the plague, and remarks on the means for its prevention, iv. 364.

SAPONARIA, botanical description and medical qualities of, xv. 263.

SATTERLEY, Dr. case of hydrophobia, xxxi. 166.

SAUCEROTTE, M. case of preternatural growth of the bones, v. 491.

SAUMEREZ, Mr. remarks on generation, and on the principle of life, ii. 242, 321; notice of his new system of physiology, iii. 184; observations on the functions of the liver, spleen, pancreas, and thyroid gland, xvi. 289; review of his principles of physical and physiological science, xxviii. 304, 330, 414; his oration at the London Medical Society, 497.

SAUNDERS, Dr. William, letters addressed to him on the subject of hepatitis, ii. 4; review of his treatise on the chemical history and medicinal powers of several of the most celebrated mineral waters, iv. 350; remarks on artificial mineral waters, viii. 488; review of his treatise on the hepatitis of India, xxii. 415.

————, Dr. James, review of his treatise on consumption, xix. 263.

SAUSSURE, Dr. de, account of his sickness and death, xvi. 470.

SAUTER, Dr. observations on the efficacy of belladonna in hydrophobia, vi. 93; description of his mode of treating fractures, xxxii. 177; of his improved instrument for the extraction of polypi from the uterus, 265.

SAVAGE, account of the discovery and education of one found at Aveyron, xviii. 376.

SAVAKESI, Dr. observations on the Egyptian ophthalmy, vi. 354; strictures on the theories of the different kinds of ophthalmy, vii. 214; review of his treatise on the Egyptian ophthalmy, viii. 555.

SAVARY, Mr. historical researches on pemphigus, xxxii. 281.

SAVINE, observations respecting its utility in gout, xxv. 544.

SAW, remarks on the advantages of that of Mr. Hey, xii. 378; description of a new circular one, xxxiii. 189.

SAWREY, Mr. review of his treatise on venereal diseases, ix. 571; observations on a recently-discovered membrane in the eye, and on fistula lachrymalis, xvii. 283, 527; review of his account of the life and writings of Dr. Marshall, xxxiii. 54, 139.

SAYER, Dr. T. B. account of worms found in the vagina of a woman, xviii. 379.

SCABIOUS, its botanical description and medicinal qualities, xi. 532.

SCALDS, observations on the treatment of, iii. 206, 296; success of the stimulating plan in the treatment of, v. 54; observations on the treatment of, 236; remarks on the use of cold water in, viii. 443.—*See* Burns.

SCAMMEL, Mr. case of secondary small-pox, xvii. 546; case of fracture of the cranium, xviii. 129.

SCAMMONY, chemical analysis of, xxv. 156.

SCANDIX, its botanical description and medicinal qualities, xiii. 370.

SCARIFICATOR, description of a new one, i. 113; proposals for a new form of the, xxiv. 177.

SCARIFYING, observations on the efficacy of, x. 425.

SCARLATINA, observations respecting the beneficial agency of cold water in the cure of, iii. 556; remarks on the treatment of, ix. 249; review of a treatise on, x. 487; efficacy of cold water in the treatment of, with several cases, xi. 27; observations on the utility of oxy-muriatic acid in the treatment of, 289; description of the eruption in, xiv. 366; considerations respecting its secondary occurrence, xv. 489; remarks on its character and treatment, 492; on the use of active stimulants in the treatment of, 493; on the use of carbonic acid gas in, xvi. 552; observations on its nature and treatment, xx. 121; essays on, xxi. 252; remarks on, especially on the anginosa, xxii. 413; further remarks on the anginosa, xxiv. 465; cases of the same, xxv. 101; utility of carbonic acid gas in the scarlatina maligna, 35; remarks on the anti-phlogistic plan of treatment, 297, 406; on the good effects of purgatives in, xxvi. 452; observations on, especially on the treatment of, xxvii. 102; xxxii. 481.

SCARPA, Professor, of Padua, observations on the structure of the bones, iv. 273; observations on several diseases of the eye, x. 561; xvi. 562; his opinion respecting the existence of pus without ulceration, xvii. 80; letters respecting the operation for spurious aneurism, 471.

SCEPTICUS, remarks on the extermination of the small-pox, ix. 356; reply to, by Credulus, xi. 154.

SCHAUB, Dr. account of his mode of obtaining prussic acid in a pure state, x. 47, 9; his experiments with charcoal in the purification of liquids, xii. 283.

SCHARD, Mr. observations respecting the use of cold affusion, iv. 25

SCHEEL, Dr. remarks on the use of frictions with oil, as a preventive of the plague, vi. 247.

SCHEELE, the chemist, his observations respecting urinary calculi, vi. 527.

SCHELLING, Dr. T. W. J. review of his work entitled Ideas, or Outlines, towards a Philosophy of Nature, iii. 384.

SCHELVER, Dr. account of his elementary doctrine of organic nature, v. 369.

SCHERER, Dr. rescued from oblivion the chemical doctrines of Mayow, i. 69; notice of his Universal Chemical Journal, 82.

SCHLEGED, Dr. observations on the use of violatricola in the cure of venereal ulcers, xiv. 431.

SCHMID, Professor, of Jena, review of his Philosophical System of Chemistry, iii. 181, 280.

SCHMIDT, Dr. his mode of preparing ammoniated iron, xii. 192; on the decomposition of sulphate of potash and soda, by lime, 575.

SCHMIDTMANN, Dr. account of a case of two years' abstinence from food and drink, iv. 86.

SCHMITT, Dr. history of a remarkable case of hydrocephalus, vi. 6; his mode of preparing æthiops antimonialis, 15.

SCHNANBERT, Dr. his mode of preparing pure gallic acid, xi. 275.

SCHRADER, Dr. observations on the generation of the earthy particles in corn, vi. 568; his discoveries respecting prussic acid, x. 95.

SCHUMACKER, Professor, critical notice of the first part of his Medical and Surgical Observations, vii. 81.

SCHUTZ, Dr. observations on the sanative powers of lime-water in diabetes, vii. 66.

SCIENCE, natural and medical, notice of Reid's archives of, iv. 73; review of Cabanis's account of the revolutions of medical, xvii. 574.—*See* MEDICINE.

SCORPION, account of the Indian remedy for its bite, xvii. 168.

SCOTT, Mr. James, case of hernia, xvii. 430; observations respecting some uses of platina, xxi. 262; observations on hæmatocele, 389; observations on hydrothorax, 443; medical sketches of practice in the navy, xxvii. 89, 189, 284, 472.

———, Mr. W. on vaccination in the Isles of France and Bourbon, xxx. 506.

———, Dr. Walter, of Stamfordham, cases of suppression of urine, successfully treated by blistering, vi. 526; remarks on the use of sulphate of zinc in intermittent fever, xi. 129; case of asthmatic paroxysm induced by the effluvium of ipecacuanha, xxiv. 233.

SCOTT, Dr. of Newport, remarks on chlorotic affections, iii. 107, 215; remarks on adhesion of the placenta, 447.

———, Dr. of the Isle of Mann, observations on the influenza of 1803, x. 529.

———, Mr. of Bromley, remarks on the use of cold application in gout, ix. 547; observations on vaccination, xv. 245.

SCROFULA, remarks on a curious case related by Professor Hufeland, and on another which terminated fatally, iv. 57; on the utility and application of galvanism in, viii. 287; ix. 132; remarks on the changes it induces in the humours of the body, explanatory of the efficacy of alkaline remedies in, 184; observations on its nature, xiv. 36; on the use of muriate of lime in, 80; observations respecting its origin, xv. 74; on its cure by the royal touch, xvii. 478; general observations on, xix. 363; on the use of mercury in the treatment of, xxiii. 494; review of an essay on the nature and origin of, xxiv. 148; remarks on the erroneousness of the opinion of Cullen respecting it, 149; doctrines of its origin from disorder of the digestive organs, 150; general observations on, xxv. 178; etymology of the term, and definition of the disease, xxxii. 499.

SCROFULARIA, botanical description and medicinal qualities of, xviii. 171.

SCROTUM, case of extraordinary enlargement of the, xv. 569.

SCURVY, observations and cases, illustrative of the efficacy of citric acid in the cure of, iv. 154; a case of, combined with severe dyspepsia, iv. 510; observations on the use of citric acid in the cure of, xii. 347; severe case of, cured by arsenic, xvi. 113; remarks on its nature and treatment, xxi. 321; observations on sea, xxiii. 233.

SEASONS, observations respecting those accompanying epidemics, xix. 317.—*See* CLIMATE.

SEA-SICKNESS, observations on its nature and causes, xix. 293, 422; xxiv. 480.

SEBALD, Mr. account of a large quantity of calculi found in the intestines of a horse, viii. 94.

SECALE CORNUTUM, botanical description and medicinal qualities of, xxxii. 90; remarks on its use in difficult parturition, 192.

SECRETION, experiments relative to the influence of the nerves on, xxxi. 486; remarks respecting its connexion with nervous and galvanic influence, xxxiii. 280, 351.

SEDUM, acre, botanical description and medicinal qualities of, xv. 265; remarks on its efficacy in epilepsy, xiv. 430.

SEEDS, account of some experiments on their germination in different gases, iv. 84.

SEGUIN, Mr. notice of his two memoirs on cinnabar, viii. 573; his remedy for intermittent fever, ix. 567; abstract of his memoir on the febrifuge principle of cinchona, xi. 215; account of his experiments on cinchona, xii. 430; his opinion respecting the efficacy of animal jelly in intermittent fever, 431; his opinion respecting fermentation, xvii. 198.

SELIG, Dr. observations on the efficacy of the extract of carduus benedictus in catarrhal diseases, vi. 370.

SELTZER-WATER, observations on its efficacy in alleviating the nausea attending pregnancy, i. 284.

SEMPERVIVUM, botanical description and medicinal qualities of, xvi. 75.

SEMPLE, Mr. observations on the effects of diseases of the gastric system on ulcers, xxviii. 200; on the utility of bleeding in ischuria, 454; cases of abscess of the liver, xxxi. 8; case of disease of the liver cured by mercury, 467; case of abscess of the liver, xxxii. 370.

SENEBIER, Mr. remarks on his experiments and observations on the germination of seeds in various gases, vii. 470.

SENECIO, botanical description and medicinal qualities of, xix. 360.

SENEKA, remarks on its efficacy in croup, i. 83.

SENEX, observations on the treatment of scarlatina anginosa, xxv. 298.

SENSATION, reflections relative to that of the head when separated from the trunk, i. 51; remarks on the effects of galvanism on, vii. 530; a new theory of, xxv. 469; xxvi. 89, 353, 433; remarks on it, 284; observations on some doctrines respecting, xxviii. 13; remarks on morbid, 441; observations on taste and flavour, 457.—*See* Life.

SENTER, Dr. history of a case of vomiting of urine, xxxi. 493.

SEPTIC FLUIDS, reflections on their affinities and relations to other bodies, ii. 180.—*See* Contagion, *and* Miasma.

SERINGAPATAM, account of a form of fever prevalent at, xxxiv. 244.

SERGEANT, Mr. observations on vaccine inoculation, iv. 565.

SERNEY, Dr. review of his treatise on inflammation, especially of the eye, xxv. 167.

SERPENTS, on the efficacy of the balm of Mecca against the effects of the bites of, i. 34; on the odour of, vi. 345; on the fatal effects of the bites of those of Martinique, iv. 293; on the efficacy of the juice of the *vejuco* or *guaco*, against the effects of the bites of, vii. 478; case of the efficacy of volatile alkali for the bite of, xi. 332, 334; observations on the poison of, 481; the *serpe nero*, (*coluber natrix*, Lin.) its disposition to suck cattle, xiii. 287; general observations on the poison of, xxiv. 391; case of fatal consequences of the bite of the rattle-snake, ibid.; remedy for the bite of the scorpion, xvii. 168; case of the effects of the bite of a rattle-snake, 398.

SERRATULA, botanical description and medicinal qualities of, xix. 260.

SERRES, M. observations on entero-mesenteric fever, xxxi. 326; observations on the eyes of insects, xxxii. 314, 400, 500.

SESASMUM, (the *sezegery*,) observations on its efficacy in the dysentery of the West Indies, viii. 553.

SEWALL, Dr. observations on absorption by the skin, xxxi. 80; on the diseases of the pancreas, 94; case of disease of the uterus, xxxiv. 102; on the use of arsenic in cancer, 198.

SEXUAL-DISEASES, general observations on, xxxi. 210.—*See* Genitals.

SEYBERT, Dr. account of his experiments on land and sea air, vi. 203, 359; on the atmosphere of marshes, vii. 113.

SHANN, Mr. observations on the influenza of 1803, x. 292.

SHAPTER, Dr. observations on a case of delirium, iii. 238.

SHAUKEY, Dr. observations on the utility of emetics in consumption, xix. 337.

SHAUL, Mr. remarks on spasmodic affections, iv. 47.

SHAW, Mr. John, case of hydrocephalus internus, iii. 517; his remedy for Egyptian ophthalmia, xvi. 283.

———, Dr. Hugh, case of wound in the lungs, xviii. 323.

SHEARLY, Mr. case of abscess in the chest, for which paracentesis was successfully performed, xiii. 21; case of gout, xiv. 120; observations on gun-shot wounds, xv. 97; case of peritoneal inflammation, xvi. 513; cases in surgery, xviii. 389; case of fracture of the cranium, xxi. 435.

SHEARMAN, Dr. observations on the connexion between amenorrhœa and phthisis, xxiii. 238; observation on the use of tin in worm cases, 34.

SHEARSMITH, Mr. case of encysted tumour, v. 520.

SHELDON, Mr. description of his anatomical cabinet, ii. 189.

SHELDRAKE, Mr. observations on distorted limbs, and on the means of remedying them, iv. 192; on the club-foot, 493; case of club-foot remedied by his instruments, v. 9; further observations on distorted limbs, 204; on distortion of the feet, with remarks on the Goseins described in Captain Turner's embassy to China, 456; his letter to Mr. Potts on the subject of artificial legs, vi. 446; notice of his hints to those afflicted with ruptures, x. 92.

SHEPHEARD, Mr. case of the effects of a large dose of opium, xix. 497.

SHERRILL, Dr. remarks on the utility of blood-letting and purging in palsy, xxiv. 375.

SHERWEN, Dr. observations on the medicinal qualities of digitalis, iii. 307.

SHILLITO, Mr. observations on intermittent fever, xiv. 99; observations on inflammation of the trachea, xxviii. 125.

SHOOLBRED, Mr. on vaccination in Bengal, xiii. 245, 336, 569; history of a case of hydrophobia cured by blood-letting, xxviii. 23.

SHORT, Mr. observations on the influenza of 1803, x. 101.

SHORTER, Mr. on vaccine inoculation, iii. 348.

SHUTER, Mr. description of a new scarificator, xxiv. 177.

SICILY, observations on some diseases occurring at, xvii. 65; account of the medical topography of, xxviii. 240; on the state of medicine in, xxxiv. 353.

SIEBOLD, Professor, remarks on dolor faciei, vi. 158, 243.

SILEX, observations on, ix. 496.

SILVER, nitrate of, observations on its efficacy in the cure of epilepsy, ii. 70; on its efficacy in various diseases, xxxi. 148.—*See* ARGENTUM NITRATUM.

SIMMONS, Mr. W. of Manchester, remarks on Mr. White's treatment of sphacelus, ii. 13; remarks on the Cæsarean operation, 433; description of a fœtus born without brain and spinal marrow, iv. 190; case of enlarged clitoris, v. 1; on the efficacy of arsenic in the treatment of cancer, 251; vi. 31; observations on cow-pox, v. 134; reply to Mr. Syer's remarks on his case of monstrosity, 254; observations on the efficacy of digitalis in many surgical cases, v. 133; on the efficacy of tincture of tobacco in gleet, vii. 57; observations on scrofula, and remarks on the doubtful efficacy of muriate of lime in that disease, 58; observations on the use of the plaster bandage in the cure of ulcers, ix. 113; on the efficacy of soda, employed externally and internally, in some severe ulcers, 267; observations on select subjects in surgery, 197; case of abscess of the liver, x. 1; observations on the incontraction of an artery, 2; case of strangulated hernia during pregnancy, 4; case of wound of the trachea, 5; case of ulcer of the leg, 8; case of early cataract, 16; continuation of his observations on select subjects in surgery, xiii. 7, 97, 289, 385; reply to Chirurgus, xiv. 103; observations on select subjects in surgery, 481; continuation of his observations on select subjects in surgery, xv. 1; observations on the utility of the adhesive strap in the cure of ulcers, xx. 255; apology for the cutting gorget, xxii. 339; observations on the medical use of spider's-web, 457; on ophthalmia, ibid.; observations on the use of the cutting gorget, xxiii. 238; on the effects of arsenic in cancer, xxviii. 118.

SIMMONS, Dr. notice of the eighth volume of his medical facts and observations, iii. 463.

SIMONS, Dr. Benjamin, notice of his work on the bones, viii. 279; on the distillation of vinegar, iv. 411.

SIMPSON, Mr. of Bristol, observations on the influenza of 1803, x. 101; his evidence in favour of vaccination before a Committee of the House of Commons, vii. 493; viii. 25; on the use of nitre in sphacelus, xiii. 324; case of prolapsus of the uterus, xiv. 98; case of ulcer of the leg, and cases of secondary small-pox, xv. 217.

———, Mr. of Skipton, case of compound fracture, x. 316; cases of sphacelus, xiii. 324.

SIMS, Dr. James, remarks on the Cæsarean operation, ii. 433; on the use of pure ammonia during pregnancy, v. 207; observations on the mode of delivery in certain cases of arm presentation, vii. 481; his observations respecting cow-pox, xiv. 480; sketch of a new theory of cow-pox, xv. 57; remarks on contagious diseases, 59.

———, Dr. Robert, biography of, xxvii. 437.

SIMSON, Professor, account of his medical dissertations, xxiv. 64.

SINAPIS, botanical description and medicinal qualities of, xviii. 267.

SINCLAIR, Sir John, bart. account of Col. Martin's mode of relieving himself from stone in the bladder, i. 120; observations on health and longevity, xiii. 527; observations on calculous complaints, xxviii. 36.

SINGER, Mr. account of his lectures on electricity, xxv. 280.

SINGULTUS, history of a severe case of, xiv. 514.

SINUS, maxillary, case of inflammation in the, ix. 423.

SIROCCO, (the wind,) observations respecting it, xix. 116; xxiv. 353, 403, 407.

SIRUM, botanical description and medicinal qualities of, xii. 365.

SIVVENS, observations relative to the history and description of, xviii. 82.

SKELETON, account of a fossil human one, found at Guadaloupe, xxxi. 338, 432.—*See* Bones.

SKEY, Dr. his evidence in favour of vaccination before a Committee of the House of Commons, viii. 24.

SKIN, remarks on the benefit derived from wearing flannel in contact with it, i. 39; case of change of colour of it from white to black in an adult female, xxv. 24; observations on a change of colour of it, xxviii. 508; observations on its structure and functions, 458; review of a treatise on diseases of the, xxxi. 412; case of change of colour of it from white to black, xxxiii. 73.—*See* Cutaneous Absorption, Affections, Diseases, *and* Eruptions.

SKINNER, Dr. J. observations on the utility of a new history of medicine, xxiv. 308; account of the plague at Malta, xxxiii. 477; observations on the use of friction with oil as a preventive of contagion, fever, &c. 482.

SKIRMSHIRE, Dr. three cases of fractured skull, iii. 23.

————, Mr. remarks on the prevalence of scarlet fever, and on its treatment, vi. 420; chemical observations on fæcula, xxviii. 568.

SKULL.—*See* Cranium.

S. L. remarks on the division of diseases into general and local, xxxi. 185.

SLEEP, physiological reflections on, xxi. 501; xxxiv. 279.

S. M. observations on vaccination, xiv. 140, 310; xviii. 428; account of the London Medical Benevolent Society, xxix. 471; queries respecting cancer, xxv. 117.

SMALL-POX, curious mode of inoculating it, ii. 468; its coincidence with measles exemplified in two cases, 572; account of the first introduction of inoculation of it into Great Britain, with the notice of a pamphlet written on the occasion, by Dr. Pierce Dod, iv. 7; cases of, combined with measles, 28; 67; case of secondary, both taken by infection, v. 403; cases of, affecting both mother and her new-born infant, 536; remarks on the probability of the extermination of the, ix. 356; reply to those remarks, 413; remarks on its extermination, xi 362; miscellaneous remarks on, 317; remarks on the identity of its source with the cow-pox, 326; cases of, subsequent to cow-pox, xii. 81, 264; case of secondary, 396; observations on its occurrence after cow-pox, 399; on the local effects of inoculation with, after cow-pox, 439; observations on Dr. Rollo's cases of its effects after vaccination, 531; account of two cases in Fuller's-rents, 574; its propagation suspended by the Harmattan wind, xiii. 1; cases of, induced by inoculation after cow-pox, 38; several cases occurring after cow-pox, and the causes assigned for their occurrences, 49; insusceptibility to it at particular periods, 51; formerly destroyed one-sixth of the population of Ceylon, 124; cases of insusceptibility to, 165; after cow-pox, 268; Mr. Goldson's work on, 268; Mr. Hunter's diagnostic character of, 400; not always present, ibid.; case of, after cow-pox, 518; cause of their union, 519; case of infection with, from a corpse buried twelve years, 198; case of, after cow-pox, 308; cases of, after cow-pox, 353, 536; case of secondary, 537; on its identity with cow-pox, xiv. 48; defence of it, 81; report of hospital for, in 1805, 194; case of secondary, 219; case of, after cow-pox, 246; comparison between it and cow-pox, xv. 301; case of secondary, 432; observations on confluent, 532; its prevalence in the vicinity of Stogarsey, in 1803, xvi. 23; observations on confluent, 62; general observations on, 181, 247; case of, after vaccination, 473; case of secondary, xvii. 137; two cases of, after cow-pox, 233, 365; case of, in the fœtus, 470; observations respecting its fatality, 512; case of, after vaccination, 514; case of secondary, 546; miscellaneous observations on, xviii. 23; cases of, in the fœtus, 177; case of secondary, 315; various observations on, 384, 566; xix. 186; on the use of cold affusion in the treatment of, 272; three cases, after cow-pox, xx. 61, 258, 259; remarks on its prevalence in India, 283; case of, after cow-pox, xxii. 88; two cases in fœtuses, 242; case of, after vaccination, 478; account of its ravages in Mexico, xxv. 239; report of the hospital for, 168; cases of secondary, xxii. 86, 430; arguments against interfering with the inoculation of, xxiii. 27; case of secondary, 123; cases of, after vaccination, xxvi. 83, 177, 249, 378; observations on the contagious property of the inoculated, x. 117; cases of secondary, 423; xxvii. 111; account of its ravages in Ceylon, prior to vaccination, xxviii. 72; case of, after vaccination, 111, 281; case of secondary, 456; case of, after vaccination, xxx. 33; account of some juridical proceedings relating to inoculation with the, xxxiv. 82, 168; extract from Mr. Moore's history of, 121; story of its being communicated by a body buried thirty years, 325; observations on its analogy to cow-pox, 295.

SMART, Mr. observations on the influenza of 1803, x. 393, 397; case of severe inflammation from vaccination, xvii. 156.

SMELL, physiological observations on, vi. 339; xxviii. 457.

SMELLIE, Dr. observations on the use of adhesive straps in the cure of ulcers, xx. 322.

SMERDON, Mr. observations relative to Sir W. Adams's publication on the cataract and the Egyptian ophthalmia, xxxii. 486; reflections on the functions of the nervous system, xxxiii. 23, 82, 276, 351, 456.

SMITH, Dr. A. cases of ischuria, xxiv. 381.

———, Dr. Edward, pathological observations, xv. 564; history of an extraordinary disease of the stomach, xix. 157.

———, Dr. T. observations on the paper of T. Y. xviii. 159; case of hæmoptœ, 320.

———, Mr. Thomas, observations on the cutting gorget, xxii. 224; a new theory of sensation, xxv. 469; xxvi. 89, 353, 433; observations on lithotomy, xxvi. 291.

———, Mr. J. case of wounded brachial artery, xii. 339; translation of Dr. Heberden's treatise on infantile diseases, xiv. 374; observations on vaccination, 60.

———, Mr. W. case of inversion of the uterus, vi. 503.

———, Mr. Robert, observations on the extirpation of diseased testicles, xxviii. 265.

———, Mr. of Chertsey, description of an improved trephine, x. 239; case of amaurosis successfully treated by electricity, xi. 135.

———, Mr. of Biddeford, observations on the influenza of 1803, x. 103.

SMITH, Mr. of Limehouse, case of stricture of the œsophagus, ix. 552.

———, Mr. H. observations on scarlatina, xxvii. 102.

———, of London, account of his flexible metallic bougies, vii. 238; xii. 276; xvii. 460; xviii. 243.

SMITHSON, Mr. observations on zeolite, xxv. 361.

SMYRNIUM, botanical description and medicinal qualities, xii. 554.

SMYTH, Dr. J. Carmichael, remarks on the hospital reports respecting his mode of destroying contagious miasmata by acid fumigation, xv. 283.

SNAILS, (*helix pomatica,*) their application advised to scrofulous ulcers, ii. 175; and for the cure of hernia, xxiv. 18.

SNOW, Mr. observation on the use of tartar emetic in ague, xii. 341; case of fractured skull, xiii. 295.

SOAP, account of a mercurial, ii. 582.

———, a mode of rendering it perfectly soluble, iii. 270.

SOCIETIES: Transactions of, &c.

SOCIETY, *Academy of Natural History*, at Berlin; account of the contents of the second vol. of their Transactions, iii. 264; account of the earthy particles found in corn, by Mr. Schrader, vi. 568; on some effects of arsenic on the human body, by Professor Pfaff, xvi. 95.

———, *Academy of Sciences*, at Erfurt; observations on cow-pox and small-pox inoculation, by Mr. Buchner, xvi. 478.

———, *Academy of Sciences*, at Stockholm; a memoir on the use of tar-water in venereal complaints, by Dr. Acharian, ix. 582.

———, *of Arcueil;* observations on the decomposition of some vegetable and animal substances by heat, by M. Gay-Lussac, xxv. 508.

———, *of Emulation*, at Abbeville; observations on some diseases of elm-trees, v. 492.

———, *Galvanic*, of Paris; observations respecting the influence of galvanism on the blood, ix. 291; on some experiments on frogs, by M. M. Nauche and Laporen, 487; experiments relative to the decomposition of muriatic acid, xv. 294; on the means of augmenting the action of the voltaic pile, 487.

———, *Geological;* some transactions of, in the year 1812, xxviii. 73, 235; xxix. 247.

SOCIETY, *Kirwanian;* account of its establishment, xxvii. 518; observations on malic acid, by Mr. Donovan, xxix. 173.

——————, *Linnean;* a memoir on the physiology of the egg, by Dr. Paris, xxiv. 164; observations on the genus *callitriche*, by Mr. Schmulz, xxix. 174; various papers, 428, 509; various transactions, xxx. 82, 250; xxxii. 75.

——————, *Lincolnshire Benevolent Medical;* account of the anniversary meeting in 1810, xxiv. 173.

——————, *Medico-Chirurgical*, of London; account of its institution, xiv. 191; analysis of the first volume of the Transactions of the, xxii. 65; containing a case of aneurism of the carotid artery, by Mr. Astley Cooper, 67; case of violent cough, cured by a preparation of iron, by Dr. Stanger, 70; observations on the treatment of hooping-cough, by Dr. Richard Pearson, 71; on a diminution (in consequence of disease) of the area of the aperture, by which the left auricle of the heart communicates with the ventricle of the same side, by Mr. Abernethy, ibid.; an account of a peculiar disease of the heart, by Sir David Dundas, 73; on the gelatine of the blood, by Dr. Bostock, 74; account of the effects produced by a large quantity of laudanum taken internally, by Dr. Marcet, 78; case of exposure to the vapour of burning charcoal, by Dr. Babington, 152; case of lithotomy, by Mr. Forster, 155; on gouty concretions, by Mr. James Moore, ibid.; case of artificial dilatation of the female urethra, by Mr. Thomas, ibid.; case of hydrophobia, by Dr. Marcet, 157; two cases of sudden death, with the appearances on dissection, by Mr. Chevalier, 158; case of intus-susceptio, with remarks, by Sir W. Blizard, ibid.; description of two mu cles surrounding the membranous part of the urethra, by Mr. James Wilson, 159; case of tumor in the brain, with remarks on the propagation of nervous influence, 161; case of a fœtus found in the abdomen of a boy, by Mr. William George Young, 166; Transactions of, vol. ii. containing a case of aneurism by anastomosis, by Mr. Travers, xxviii. 56; a case of hydrocephalus internus in a precocious girl, by Mr. Coote, 58; case of a precocious girl, by Dr. Wall, ibid.; case of hernia cerebri, by Mr. Burrow, 59; case of wound of the heart, by Mr. Heatherton, ibid.; case of extraordinary enlargement of the right lower extremity, by Mr. Chevalier, 60; case of lithotomy, with observations, by Mr. Chevalier, 61; case of erythema, not mercurial, by Dr. Marcet, ibid.; case of singular nervous affection, by Dr. Marcet, 62; chemical account of various dropsical fluids, by Dr. Marcet, ibid.; cases of tumor of the spleen, by Dr. Bree, 63; case of a knife lodging for several years in the back of a man, by Mr. Bush, ibid; case of fracture of the occipital bone, by Dr. A C. Hutchinson, ibid.; observations on the urine voided in diabetes, by Dr. Henry, ibid.; case of a woman who swallowed a large dose of arsenic, by Dr. Roget, 63; observations on the serum of the blood, by Dr. Bostock, 66; on the mercurial plan of treating dysentery, by Dr. Ferguson, 67; a case of dysphagia, by Mr. Armiger, 151; account of the dissection of a limb, on which the operation for popliteal aneurism had been performed seven years previously, by Mr. Astley Cooper, 152; a case of hydatid in the brain, by Mr. Morrat, 154; observations on tumors in the pelvis, occasioning difficult parturition, 154; case of fractured cranium, by Mr. Creagh, ibid.; case of worms in the bladder, by Mr. Lawrence, 155; case of tetanus, by Mr. Harkness, ibid.; case of tetanus, by Sir W. Blizard, ibid.; observations on the use of arsenic after the bites of venomous animals, by Mr. Ireland, ibid.; Transactions of, vol. v. xxxiii. 60; case of abscess in the brain, by Dr. Denmark, ibid.; observations on nyctalopia, by Mr. Bampfield, 63; case of laceration of the stomach and duodenum by vomiting, by Mr. Chevalier, 64; on contraction after burns, by Mr. Henry Earle, 65; case of a child retained within the abdomen fifty-two years, by Dr. Cherton, ibid.; on some diseases of the toes and fingers, by Mr. Wardrop, 151; on some of the causes which destroy the fœtus in the uterus, by Dr. Stewart, ibid.; case of a child born without a brain, by Mr. Lawrence, ibid.; case of spinal malformation, by Mr. Lawrence, ibid.

SOCIETY, *Medical*, of Brussels; observations on the medicinal qualities of the toxicodendron, by Dr. Van Mons, vi. 272.

——————, *Medico-Chirurgical*, of Parma; observations on the use of cannabina as a vermifuge, by Prof. Colla, xxiv. 517.

——————, *for promoting Medical and Surgical Knowledge*, London; analysis of the second volume of the Transactions of the, containing an account of a case in which death was brought on by a hæmorrhage from the liver, by Sir Gilbert Blane, iv. 65; observations on croup, as it appeared in Chesham, by Mr. Rumsey, 67; an account of two cases, shewing the existence of the small pox and the measles in the same person at the same time, by Dr. P. Russel, ibid.; observations on the management of cases in which the face of the child presents towards the os pubis, by Dr. John Clarke, 163; a case of pregnancy, where the ovum had become diseased, and was entirely filled with small hydatids, by Sir Everard Home, 165; on the use of the application of gastric juice to sores, by Dr. Harness, 167.

SOCIETY, *Medical and Physical*, of Bourdeaux; programme of the memoir which obtained the prize given by that society in the year 1801, viii. 280; on the sensibility of plants, by M. Dutrouil, ix. 291.

————, *Medical*, of London; analysis of the fifth volume of the Memoirs of, ii. 29; containing a case of hydrophobia, by Mr. Gaitskell, 30; observations on the efficacy of the zanthoxylon, by Thomas Henry, M. D. with an account of the tree, by Mr. Chamberlaine, 30, 31; observations on the lithontripic power of the muriatic acid, by Mr. Copland, 34; experiments on the external use of the tartarized antimony, by Mr. Hutchinson, 34; case of cynanche trachealis successfully treated by Mr. H. Field, 146; observations on human intestinal worms, by Dr. R. Hooper, 149; annual oration for the year 1802, by Dr. James Sims, vii. 297; prize medals for the year 1802, 300; list of officers, &c. for the year 1803, ix 591; the sixth volume of the Transactions of the, xv. 377, containing a sketch of the similarity of the ancient and modern opinions respecting the morbus cardiacus, by Dr. Falconer, 377; a case of angina pectoris, by Dr. Black, 380; a case of hydrocephalus internus terminating favourably, by Mr. Gapper, 380; a case of a blue boy, 381; a case of obstinate hepatic disease, by Dr. J. C. Lettsom, 381; a case of a remarkable and successful termination of a scrotal hernia, by Mr. Lee, 331; a case of croup successfully treated, by Mr. J. Smith, 382; on the origin of the cow-pox, by Dr. Marshall, 382; case of extra-uterine fœtus, by Dr. A. Fothergill, 385; case of inverted uterus after parturition, by Mr. Dyson, 385; case of extraordinary disease found in the left cavity of the thorax, by Mr. Carden, 385; history and dissection of a case of intestinal ulceration, by Mr. Field, 386; history of a case of peculiar morbid appearances in the heart, 386; case of a wound in the peroneal artery, by Mr. Cam, 563; observations on the medical use of the white oxyd of bismuth, by Dr. Marcet, 563; on the use of Bath waters in ischias, by Dr. Falconer, 565; observations on the position of patients in the operation of lythotomy, by Dr. W. Smith, 569; case of enlargement of the scrotum, by Dr. Broadbent, 569; two cases of diabetes, by Dr. Bostock, 569; on the lithontripic power of muriatic acid, by Mr. Copland, 570; anniversary meeting in 1808, the oration by Mr. Good, on the structure and physiology of plants compared with those of animals, xix. 381; anniversary meeting in 1810, xxiii. 352; Transactions of the, vol. i. part i. xxv. 56, containing a paper on medical technology, by Mr. Good, xxv. 57; biographical account of Mr. Hewson, by Dr. Lettsom, 59; history of the fatal effects from white lead, by Mr. Derring, 60; history of a case resembling hydrophobia, from the bite of a cat, 61; observations on the indiscriminate use of mercurial preparations, by Dr. Falconer, ibid.; observations on some diseases of the eye, by Mr. Ware, 161; case of suppuration of the liver, by Mr. Burns, 164; observations on the hare-lip, by Mr. Rand, 165; cases of injury from a fall, and of satyriasis, by Mr. Norris, 166; case of tic douloureux, by Dr. Anthony Fothergill, 167; cases illustrating the effects of oil of turpentine in expelling the tape-worm, by Drs. Lettsom, Hancock, Fothergill, Birbeck, and Mr. Saner, ibid.; meeting in the year 1811, 364; oration by Mr. Blair, on the history of syphilis, ibid.; meeting in the year 1812, xxvii. 333; oration by Dr. Temple, ibid.; meeting in 1814, oration by Dr. George Rees, xxxi. 256; meeting in 1815, oration by Mr. Taunton, xxxiii. 336.

SOCIETY, *Medical*, of Massachusetts; observations on the vesicating qualities of the potatoe-fly, by Dr. Gorham, xxiii. 325; observations on the treatment of strangulated hernia, by Dr. J. C. Warren, 379; Transactions of the, vol. ii. part ii.; a dissertation on the progress of medical science in the commonwealth of Massachusetts, xxvi. 214; report on the spotted fever of the United State, 217.

————, *Medical*, of Nancy; case of maladie blue, by M. Thiebaut, x. 575; remarks on hydrophobia, by M. Valentin, xxii. 53.

————, *Medical*, of Montpellier; programme of the prizes proposed in the year 1802, ix. 91; case of a wound penetrating the abdomen and thorax, by Dr. Barthez, x. 250; case of a variety of intermittent fever, by Mr. Dumas, 427, 541; a memoir on the functions of the lungs, by Dr. Thomas, xi. 46; physiological observations on the transformations of the organs of the body, by Professor Dumas, xvi. 301, 451.

SOCIETY, *Medical,* of New York; enquiries respecting the origin and means of prevention of the epidemic fever of the United States, i. 199; Transactions, in the years 1809 and 1810, xxvi. 421.

————, *Medical,* of Paris; a memoir on the medicinal properties of oxygen, by M. Fournier, ii. 38; observations on the utility of compresses, moistened with cold water, and applied to the abdomen in strangulated hernia, by M. Lombart, 46; case of distortion of the abdomen, from the use of stays, by M. Lombart, 47; case of abscess of the liver, by M. Lombart, 48; account of the discharge of an insect from the nose, by M. Lombart, 49; case of fistula in ano, by M. Lombart, 154; case of cholera morbus, by M. Lombart, 155; general remarks on aliments produced by the different classes of the animal kingdom, and their influence on the human body, by M. J. J. Virey, iii. 65, 153, 249; case of puriform destruction of a great part of the brain, by M. Chizeau, 70; instance of family disposition to hydrocephalus, 71; on the utility of carbonate of potash in puerperal fever, by M. M. Allan, Deyeux, Gilbert, and Lafisse, 80, 165, 264; observations on a new species of salt, by M. Chaussier, 82; analysis of the cassia senna, by M. Bouillon Lagrange, 85; observations on the cholic that prevailed at Madrid, by M. Alibert, 167; case of leprosy, by M. M. Alibert, Fourcroy, and Halle, 167; experiments on the agency of medicinal substances applied as friction, by M. M. Alibert and Dumeril, ibid; case of disorganization of several of the abdominal viscera, from a kick from a horse, without laceration of the skin, by M. Larrey, 168; observations on the effects of alkaline substances in the treatment of fistula lachrymalis, by M. Pajot des Charmes, ibid; case of paralysis of the exterior muscles of the hand and fingers, accompanied with separation of the bones of the metacarpus, by M. Bourguet, 253; observations on the effects of acetic ether applied by friction in rheumatic affections, by M. Martin, iv. 369; on epizootic disease, by M. Buniva, 373; observations respecting the cause of rickets, by Dr. Borda, vi. 191; on the efficacy of acetic ether used by way of friction in the cure of rheumatism, by M. Desparanges, 273; on the mode of preserving anatomical preparations, by Professor Chaussier, xx. 274; observations on the effects of lightning, by M. Coquart, 277; remarkable case of mental derangement, ibid.; observations on epilepsy, by M. Portal, 377; observations on strangulated scrotal hernia, by M. Fardeau, ix. 260; account of a leech adhering to the œsophagus, by M. Larrey, 262; report on the disease termed the *gripe*, prevalent in Paris in the year 1803, 419; observations on the internal use of mercurial ointment, in the cure of the venereal disease, by M. Terras, 487; on the use of gelatine in intermittent fevers, by M. Gilbert, x. 323; on dropsy of the ovarium and fallopian tubes, causing a displacement of the uterus, by Dr. M. Voisier, xi. 73; observations on the Guinea-worm, by M. Larrey, xii. 434; case of hydrophobia, by M. Sabatier, xvii. 218, 344; two cases of closure of the glottis, by M. Delorme, xxii. 140.

SOCIETY, *Medical School,* of Paris; case of fœtus without head or lower extremities, by M. Dupuytren, viii. 382; case of ovarian gestation, ix. 56; account of the successful division of the symphysis pubis, by M. Mansey, 57; observations on the umbilico-mesenteric vessels, by M. Chaussier, 170; account of some calculi extracted from the fossa navicularis, by M. Dumeril, 268; case of tinea, by M. Baysillance, x. 542; experiments on the febrifuge principle of cinchona, by M. Seguin, xi. 215.

————, *Medical,* of Philadelphia; the anniversary oration of 1806, on quarantines, by Dr. Caldwell, xviii. 111.

————, *Medical,* of South Carolina; an account of several cases of acute bilious fever successfully treated by nitric acid, by Dr. Aule, xix. 106.

————, *Medical,* of Dublin; account of the institution of, xxxiv. 81.

————, *National Institute,* of France; observations on the dilatation of the uterus, by M. Penon, vi. 278; case of morbid elongation of the tongue, by M. Lassus, 353, 435, 522; observations relative to eudiometry, by M. Berthollet, 376; observations on vegetable anatomy, by M. Mirbel, vii. 557; a memoir on the fulminating mercury, by M. Berthollet, viii. 190; a memoir on two non-descript oviparous quadrupeds, by M. Lacepede, 283; observations on a case of universal anchylosis, by M. Percy, 284; case of polyphagy, by M. Percy, ibid.; researches on the voltaic pile, by M. Guyton, 478; on the composition of œthiops mineral, by M. Sequin, 573; experiments on mercurial salts, by M. Fourcroy, ix. 9; on the use of animal jelly in intermittent fevers, by M. Seguin, 567; account of extraordinary power of resistance of heat, by a Spaniard, x. 380; a memoir on virous fermentation, by M. Thenard, xi. 411; on the febrifuge principle of cinchona, by M. Seguin, xii. 430; on the efficacy of animal jelly in the treatment of intermittent fevers, by M. Halle, 431; a description of the cahalot and crocodile, by M. Zenon, xvii. 485; observations on the soporific effects of the effluvium of saffron, by M. Le Sage, xviii. 288; an account of an analysis of the acid contained in the sweat, urine, and milk, by M. Thenard, xix. 124; experiments on the division of the eighth pair of nerves, by M. M. Dupuytren and Duprey, xx. 98; a memoir on muriatic ether, by M. Thenard, 125; experiments on the decomposition of the earths by electricity, 569; considerations on the nature and treatment of hereditary or family diseases, by M. Portal, xxi. 229, 281; an enquiry into the action of some vegetables upon the spinal marrow, by M. Magendie, xxii. 96; report of a memoir on the tetanus rabiensis, by M. Girard, xxiii. 41; report of a treatise on champignons, by Dr. Paulet, 68; report on the progress of medical science in the year 1809, 451; a memoir on the organs of absorption in mammiferous animals, by M. Magendie, xxv. 44; chemical report in the year 1810, 362; observations on making an endowment for sending physicians to other countries, by M. Oddier, xxvi. 420; account of its proceedings relative to medical science in the year 1811, xxvii. 374; report on botany and vegetable physiology, xxx. 251; on zoology, anatomy, and animal physiology, in 1812, 429, 517; report on the memoir of M. Magendie on vomiting, xxxii. 518; xxxiv. 524; proceedings in anatomy, physiology, and medicine, in the year 1814, xxxiii. 396.

SOCIETY, *Philosophical*, of London; meeting in the year 1812, xxviii. 422; various proceedings of, xxix. 81, 336, 436, 521.

————————, *of Pharmacy*, at Paris; report on the memoirs preferred for the prizes in the year 1809, xxiv. 224.

————————, *Philomatic*, of Paris; plans for an anatomical nomenclature, by M. M. Dumeril, Chaussier, and Dumas, ii. 479; report on Dr. Sœmmering's observations on the retina, by M. M. L'Eveille, Larrey, and Dumeril, ibid.; case of imperforate anus, ibid.; case of a fœtus having but one eye, ibid.; case of suppuration of the pericardium, by M. L'Eveille, 480; case of cerebral hernia, by M. Halle, ibid.; case of a fœtus without either head or heart, by M. Cuvier, ibid.; case of a fœtus contained in the ovarium, by M. L'Eveille, ibid.; account of the dissections of several apes, by M. Brogniart, ibid.; observations on the hearing faculty of whales, by M. Cuvier, ibid.; observations respecting the organs of generation of the *julus complanatus*, 481; account of the performance of the Cæsarean operation, by M. L'Eveille, ibid.; case of aneurism of the aorta, by the same, ibid.; case of extra-uterine conception, by M. Lacroix, ibid.; case of tetanus, by M. L'Eveille, ibid.; observations respecting the formation of preternatural joints, by M. Dumeril, 482; case of caries of the femur, by M. Larrey, ibid.; observations on a concrete volatile oil, by M. Vauquelin, 88; observations on some chemical combinations of potash, by M. Van Mons, ibid.; observations on the gluten of wheat treated by acids, by M. M. Vauquelin and Brogniart, ibid.; on the effluvia of odorous bodies, by M. Brogniart, 89; observations on camphoric acid, by M. Bouillon Lagrange, ibid.; observations on the essential particles of compound bodies, by M. Lamarck, ibid.; account of the analysis of snow-water, by M. Lacroix, 90; on the extraction of soda from sea-salt, by M. Lelievre, ibid.; on the preservation of water in sea voyages, ibid.; on the antiseptic property of beer, by M. Chauteau, ibid.; on the extraction of the oil of juniper, by M. Bouvier, ibid.; on the preservation of the colours of flowers, by M. Hauy, 91; sketch of the history of the healing art, by M. Halle, 172; on the difference between the acetic and acetous acids, by M. M. Adet and Chaptal, ibid.; discovery of chrome and chronic acid, by M. Vauquelin, 173; analysis of the ruby, by the same, ibid.; discovery of the chrome in the emerald, by the same, ibid.; experiments on the extractive principle of vegetables, by the same, ibid.; analysis of a gouty concretion, by M. M. Vauquelin and Fourcroy, 174; analysis of the urine, by M. Fourcroy, ibid.; on the manner of fabricating vessels of porous earth in Spain for the purification of water, by M. Lasterie, ibid.; observations on the production of carbon by decomposing carbonate of lime by phosphorus, by M. M. Vauquelin and Brogniart, 374; observations on the characters to be adopted in describing mineral substances, by M. Brogniart, 375; observations on the pudding-stones of the Alps, by the same, ibid; case of idiopathic atrophy, by M. Halle, ibid.; observations on the circulation of the blood in animals in whom that fluid is white, by M. Cuvier, 376; observations on the nutrition of insects, by the same, ibid.; a memoir on mildew, by M. Chautran, iv. 372; on the movements of odorous substances placed on water, by M. Prevost, vii. 592; on the difference between the crocodiles of the ancient and of the new world, by M. Cuvier, 575; account of some blue oxydated iron, by M. Vauquelin, 479; analysis of the baracites, by the same, 92; experiments on gum kino, by the same, xi. 277.

SOCIETY, *Royal*, of London; account of Dr. Thomson's history of it, xxviii. 314; experiments on guaiacum, by Mr. Brande, xv. 294; on artificial tanning, by Mr. Hatchett, 295; account of a species of worm-shell found on the coast of Numatia, by Mr. Griffiths, 391; observations on a particular affection of the prostate gland, by Sir E. Home, 487; the award of the Copleyan medal in the year 1807, to Mr. Knight, xvii. 196; the Bakerian lecture in the year 1808, on the decomposition of fixed alkalies, with some remarks on electrical agency in chemical phenomena, xix. 93, 190; the Croonian lecture for 1808, by Mr. Carlisle, comprising a physiological view of the circulation of the blood, and of the influence of the nerves on the muscular fibre, 191; a memoir on the structure and uses of the spleen, by Sir E. Home, xx. 211; electrical experiments on the decomposition of the earths, by Sir H. Davy, 383; experiments relative to the chemical phenomena attending respiration, by Messrs. Allen and Pepys, xxii. 48; observations on the chemical composition of urinary calculi, by Mr. Brande, 49; observations on the influence of electricity on animal secretions, by Dr. Wollaston, 174; a memoir on the boa upas poison, by M. Delieie, xxiv. 174; an account of a calculus taken from the bladder after death, by Sir James Earle, 185; observations on the effect of magnesia in preventing the formation of uric acid, by Mr. Brande, 203; the Croonian lecture in the year 1810, by Dr. Wollaston, 485; observations on some of the combinations of oxy-muriatic gas and oxygen, and on the chemical relation of these principles to inflammable bodies, by Sir H. Davy, xxv. 83; the Bakerian lecture in the year 1811 on some chemical combinations of oxygen, by Sir H. Davy, 180; the Croonian lecture, by Mr. Brodie, on the influence of respiration on the temperature of animals, ibid.; observations on cystic oxide, by Dr. Wollaston, 254; observations on animal heat, by Mr. Macartney, 278; observations and experiments on pus, by Dr. George Pearson, 326; on the connexion of the stomach with the spleen and bladder, by Sir E. Home, 361; observations on zeolite, by Mr. Smithson, ibid; on the effects of vegetable poisons on animals, by Mr. Brodie, ibid.; on a gaseous combination of oxymuriatic gas and oxygen, by Sir H. Davy, 362; observations on luminous animals, by Mr. Macartney, 409, 512; experiments on the manner in which plants shoot forth their radicals, by Mr. Knight, 452; researches respecting the influence of the brain on the action of the heart, and on the generation of animal heat, by Mr. Brodie, xxvi. 58, 129; observations on wounds of the intestines, by Mr. Travers, x. 167; observations on vision, by Dr. Wells, 247; on a combination of oxymuriatic gas and oxygen gas, by Sir H. Davy, 298; case of nervous affection cured by compression of the carotids, by Dr. Parry, 458; on the non-existence of sugar in the blood of patients of diabetes, 462; observations on the blood, by Mr. Brande, xxvii. 73, 168; account of a vegetable wax from Brazil, by the same, 73; observations on the comet, by Sir W. Herschel, 256; on the power of distinct vision at different distances, by Mr. Campion, 257; further experiments on poisons, by Mr. Brodie, 427; observations on arsenic, by Dr. Garthshore, 518; experiments on silicated fluoric gas, and on fluoboric gas, by Mr. John Davy, xxviii. 157; on some combinations of phosphorus and sulphur, by Sir H. Davy, 157; xxx. 83; annual meeting in 1812, xxix. 172; on the black matter expectorated from the lungs, by Dr. Pearson, 506; on some fossil remains found at Brentford, by Mr. Trimmer, 507; on the formation of fat in the intestines, by Sir E. Home, ibid.; account of a woman part of whose skin is black, by the same, 508; on the alcohol of sulphur, by M. Berzelius and Dr. Marcet, 509; xxx. 79; telescopic observations, by Mr. Kater, 248; on the use of magnesia in calculous disorders, by Mr. Brande, 249; on the detonating compound of chlorine and azote, by Sir H. Davy,

424; analysis of a volcanic product, by Mr. Smithson, ibid; on the cold produced by the evaporation of sulphuret of carbon, by Dr. Marcet, 425; on the composition of fluor spar, by Sir H. Davy, ibid.; description of a sliding scale of chemical equivalents, by Dr. Wollaston, xxxi. 167; Croonian lecture, on the influence of the nervous system on muscular motion, by Mr. Brodie, 168; on a new gas, by M. Courtois, 255; further experiments on fluorine, by Sir H. Davy, 337; on the means by which vitality is supplied to the living system, by Dr. Crichton, 431; on the Indian method of oxydizing silver, by Dr. Hayne, xxxii. 74; on the effect of injuries of the brain on sensation, by Sir E. Home, 162; on the influence of the nerves on the beating of the arteries, by the same, 248; anniversary meeting in 1815, 157; on vision at different distances, by Mr. Travers, 157, 243; on the action of acids on the oxymuriate of potash, by Sir H. Davy, xxxiv. 79; account of a fœtus found within the abdomen of a child, by Dr. Philips, 80; on the nature and cause of the pulse, by Dr. Parry, 80, 163; account of the sorbic acid, by Mr. Donovan, 164; on the connexion between the vascular and extra-vascular parts of animals, by Mr. Carlisle, 165; on the effects of a large galvanic battery, by Mr. Children, ibid.

SOCIETY, *Royal*, of Edinburgh; an account of the analysis of sodalite and allanite, by Dr. Thomson, xxv. 181; xxvi. 168, 303; account of the mineralogy of Iceland, by Sir G. Mackenzie, xxv. 363; other miscellaneous proceedings, ibid.

———, *Royal*, of Gottingen; observations on the paralysis of the pupil, by Dr. Himley, vi. 275; a memoir on the yellow fever, by Dr. Guttfeldt, x. 383.

———, *Royal Humane*, of London; the prize dissertation on the means of preservation of shipwrecked mariners, by Dr. A. Fothergill, iii. 285; report of, in the year 1807, xxiii. 336.

———, *Royal Jennerian*; anniversary meeting of 1804, xi. 572; report of the meeting in the year 1806, xv. 107; report of the second meeting in the year 1806, xvi. 478; report in the year 1808, xx. 363.

———, *Royal*, of Haarlem; proceedings of, in the year 1810, xxvi. 76, 169.

———, *Vaccine*, of Paris; letter to Dr. G. Pearson on the progress of vaccination in France, iv. 251; second report of the state of vaccination in France, v. 99; report in the year 1810, xxv. 181.

SOCIETY, *Wernerian*; account of the meeting, Jan. 14, 1809, xxi. 262; observations on the geognostic relations of the rocks of the island of Arran, by Professor Jameson, xxv. 453; miscellaneous transactions, xxvi. 168; xxviii. 75; xxix. 429; xxxii. 75.

SODA, observations on its agency in digestion, and in preserving animal matter from putrefaction, ii. 383, 470; on the separation of it from sea-salt, 169, 376; the urate of, constitutes a great proportion of the matter of gouty concretions, 174; its utility externally and internally in some species of foul ulcers, ix. 267; chemical description of the urate of, 492.

SODIUM, mode of preparing it, xx. 12.

SODALITE, chemical analysis of, xxvi. 303.

SODEN, Mr. observations on hydrophobia, xvii. 432.

SOLANUM, botanical description and medicinal qualities of, xii. 134.

SOLAR and LUNAR INFLUENCE, case of periodical difficulty of breathing, supposed to be dependant on, viii. 401; its influence in the intermitting fevers in the East Indies, xxi. 75; review of a treatise on, xxvii. 398.

SOLIDAGO, botanical description and medicinal qualities of, xix. 364.

SOMERVILLE, Mr. J. account of the poisonous effects of the effluvium of the sumach on bees, xvii. 487; observations on the diuretic qualities of the pyrola umbellata, xxxiii. 507.

SONNINI, Sig. his account of the practice of midwifery in Greece, vi. 151.

SORBUS, botanical description and medicinal qualities of, xiv. 572; xvi. 168.

SOUTHALL, Mr. observations on the influenza of 1803, x. 102.

SOUTHAMS, Mr. observations on the cow-pox, xvi. 436.

SOUTHEY, Dr. review of his treatise on consumption, xxiv. 238.

SPAIN, remarks on the origin of the fever epidemic there in the year 1800, xiii. 103; remarks on the state of medicine in the latter part of the last century, xxxi. 237.

SPALDING, Dr. Lyman, observations on the fever prevalent at New Hampshire in the year 1793, iii. 568; case of apoplexy, xvii. 423; bills of mortality for Portsmouth, New-Hampshire, for the years 1806, 7, and 8, xxi. 523.

SPALLANZANI, l'Abbé, observations on the artificial impregnation of animals, i. 47.

SPARK, Dr. observations on the utility of opium in some affections of the uterus, xxx. 29, 315.

SPECIFICS, reflections on the remedies so termed, xxxii. 293.

SPECULATIONS, *medical*, reflections on, xi. 458.

SPECULUM, *oculi*, remarks on the necessity of an improvement in its form, iv. 103.

SPEECH, observations on impediments of, xiii. 450; xiv. 256; xv. 172.

SPEEDWELL, botanical description and medicinal qualities of, xi. 371.

SPEER, Dr. observations on the influenza of the year 1803, x. 405.

———, Dr. J. C. observations on the physical constitution of water, xxviii. 433.

SPENCE, Dr. observations on the efficacy of the plant sesamum in the cure of the dysentery of the West Indies, viii. 553; case of epilepsy, xviii. 355; case of consumption cured by digitalis, 511; observations on the "wind of a ball," xxviii. 142; case of disease of the stomach, xxx. 514.

SPENCER, Mr. observations on idiosyncracy, and on a case of laceration of the urethra, xxi. 485; observations on hydrocephalus, and on uterine hæmorrhage, xxiii. 190; observations on cynanche trachealis, xxvii. 44.

SPERGULA, botanical description and medicinal qualities of, xv. 267.

SPERMACETI, observations on its preparation for medical use, iii. 263, 515.

SPHACELUS.—*See* GANGRENE.

SPIDER'S-WEB, observations respecting its utility in various diseases, xxi. 353; further observations on its supposed medical qualities, xxii. 94; remarks tending to shew its inutility, 218, 369, 370.

SPIGELIA MARILANDICA, observations on its efficacy as a vermifuge, viii. 428.

SPINA-BIFIDA, history of a remarkable case, iii. 104; account of a fatal case, x. 237; a case of, which terminated fatally after the use of a ligature to the base of the tumor, xiii. 496; fatal case of, xv. 237; case of, joined with hydrocephalus, xxi. 66; observations on, xxiv. 341; xxviii. 153; case of, accompanied with a tumor at the anterior fontanelle, xxxii. 526.

SPINAL MARROW, observations on a motion in the, vi. 370; case of disease of the, xviii. 570; observations on the influence of some vegetables on the, xxii. 96; its influence on the rest of the animal economy, xxvii. 375; observations on inflammation of, and on its treatment, xxviii. 29; observations on its diseases, xxxiii. 198.

SPINE, account of some phenomena consequent on injury of the, xx. 111; observations on fracture of the, xxv. 201; case of fracture of the, xxvi. 371; observations on the use of issues in the cure of diseases of the, xxviii. 182; review of a treatise on diseases of the, xxxi. 245; case of luxation of the, 271; case of anchylosis in the neck, 307; review of a treatise on diseases of the, xxxiii. 406.

SPIRÆ, botanical description and medicinal qualities of, xvi. 171.

SPIRITS, mode of increasing the quantity of in distillation, iii. 272.

SPLEEN, observations respecting its functions, xvi. 119; dissection of a case of disease of the, 471; reflections on its functions, xvii. 161; observations on its structure and use, xx. 211; physiological observations on the, xxii. 39; case of rupture of it from external injury, xxvi. 335; case of inflammation of the, xxx. 169.

SPONGE, burned, remarks on its efficacy in the cure of bronchocele, iv. 412; xii. 9; xiii. 13; on its use in ophthalmia, xii. 233.

SPRENGEL, Prof. extracts from his history of medicine, ii. 54, 155, 278, 364, 453, 60, 160, 281, 368, 459; iii. 72, 136, 256, 356; iv 340.

SPRY, Mr. history of a remarkable case of hydrocephalus, ii. 131; observations on morbid anatomy, iii. 17; cases in, 20, 22; case of poisoning from an unknown substance, iv. 516; account of a case of hernia in a cat, v. 105; remarks on the proper tabular arrangement of diseases, 107; case of disease of the heart, xv. 386.

SPURZHEIM, Dr. exposition of Dr. Gall's system of craniology, xv. 201; review of his work on the nervous system, xxxiii. 228, 485; observations on the functions of the brain, in reply to Dr. Baillie and Sir Everard Home, xxxiv. 309.—*See* GALL.

SQUALUS, maximus, anatomical description of the, xxx. 250.

SQUIRE, Dr. observations on extraction of the placenta, iii. 409.

SQUIRREL, Mr. review of his work on cow-pox and scrofula, xiii. 465.

STABLES, Mr. observations on the influenza of 1803, x. 355; case of wound in the omentum, 356.

STACHYS, botanical description and medicinal qualities of, xvii. 563.

STAG, instance of one rendered docile, xxiii. 85.

STAHL, G. E. his doctrine respecting the relation between hypochondriacal and arthritic affections, i. 151; his doctrine of phlogiston, 64.

STANGER, Dr. case of severe cough cured by iron, xxii. 70; observations on inoculation, xiv.

STANLEY, Mr. observations on the influenza of 1803, 527.

STAPHYLOMA, observations respecting its nature and treatment, x. 566; xxv. 161, 359.—*See* Eye.

STAR, Mr. case of peritoneal inflammation, xxxiv. 443.

STEAM, description of an apparatus for applying it locally, xxxii. 18.—*See* Bath, *and* Vapour.

STEEL, Mr. observations on the influenza of 1803, x. 228.

———, Mr. A. observations respecting the torpidity of swallows, xxiv. 187.

———, mode of preventing the oxydation of, xi. 286.—*See* Iron.

STEINBECK, Mr. account of his work on cow-pox, viii. 476.

STEMACHER, observations on pharmaceutical preparations, xi. 176.

STENHOUSE, Mr. pathological observations on gout, xii. 60; on the effects of the warm bath in gout, 67.

STERN, Mr. recommends the use of the seeds of the phellandrum aquaticum in consumption, vi. 95.

STERNUM, observations on fracture of the, xviii. 200, 415.

STEVENS, Mr. G. case of aneurism of the gluteal artery, xxxiv. 508; obervations on vaccination, 20.

STEVENSON, Dr. J. observations on vaccine inoculation, vi. 121; vii. 9; account of the successful treatment of a severe case of dysphagia, viii. 35.

——————, Mr. C. observations on poisonous plants, xiv. 425.

——————, Mr. John, review of his work on morbid sensibility of the eye, xxv. 67; observations on the treatment of cataract, xxviii. 257, 358, 420; observations on the operation for artificial pupil, xxxiii. 192; case of deafness from the presence of a foreign body in the ear, 226.

STEWART, Dr. observations on vaccine inoculation, iii. 234; on the use of opium in uterine hæmorrhage, xxxii. 153.

STIZOLOBIUM, observations on its efficacy as a vermifuge, iv. 267.

STOCK, Dr. account of Dr. Rousseau's experiments on cutaneous absorption, xv. 274; observations on the effects of cold air as a remedy for some diseases, xvi. 366; extracts from his life and writings of Dr. Beddoes, xxv. 72, 259, 342.

STOMACH, account of its appearance in a person who died from taking phosphorus, i. 412; in a case of death from opium, v. 491; from drinking ardent spirits, vi. 507; in hydrophobia, xiii. 161; in gastritis, 538; in hydrophobia, xvii. 222, 350; xx. 198, 201, 209, 358; xx. 533; xxi. 58; in poisoning by opium, v. 491; xxxi. 195; in cases of poisoning by arsenic, xxii. 427; by corrosive sublimate, xxvi. 156; by arsenic, xxviii. 349, 350; xxxiv. 442; in hydrophobia, xxxi. 166; xxxiii. 317, 329; its appearance in a horse who died of tetanus, xxiv. 272; in rabies in dogs, xxv. 494; observations on inflammation of the, iii. 229; case of ulceration of the, 511; on the good effects of æther and opium in spasm of the, iv. 444; cases of disorder of, successfully treated by alkalies and absorbents, viii. 289; case of fistula of the, 361; case of extraordinary appearance of the, xiii. 164; extraordinary perforation of, ibid.; review of a treatise on its diseases, xv. 576; remarks on the same, xvi. 422; xviii. 18; remarks on its passive and active influence in diseases, xvi. 273; remarks on its diseases, 422; case of inflammation of the, 535; general remarks on its diseases, xv. 481, 573, 576; xvi. 422; observations on its functions, xvii. 163, 165; of extraordinary disease of the, xix. 157; its digestion after death supposed to be mistaken for disease during life, 170; influence of deranged action of the, on the brain, 300; several cases, illustrated by dissections, shewing the connexion between inflammation of the stomach and apoplexy, and other affections of the brain, xx. 412, 540; case of schirrus tumor of the, xxviii. 139; case of cancer of the, xxx. 337; observations on its digestion after death, xxi. 348, 426; observations on its digestive functions, xxii. 5; on its connexion with the spleen, 6; account of a man who swallowed knives, &c. 350; peculiar effects of certain species of aliment on it in some individuals, 403; observations on the connexion of derangement of its functions on other diseases, xxiii. 246; on its digestion after death, xvii. 569, 399, 428; general observations on its disorders, xxiv. 237; observations respecting the state of it in tetanus 272; observations and experiments respecting its connexion with the kidneys, xxvii. 13; case of inflammation of, from the external application of sublimate, 167; case of extraordinary capacity of the, 452; observations on the morbid anatomy of the, 494; inflammation of the, considered as the cause of hydrophobia, xxx. 201; case of cancerous affection of the, 337; singular affection from the presence of a parasitical animal, 514; on the state of in hydrophobia, xxxi. 1, 5, 281; remarks on its digestion after death, 2, 7, 77; observations on a vascular appearance of it after death, sometimes mistaken for inflammation, xxxii. 155; on the connexion of derangement of, with affections of the uterus, 327; cases of laceration of the, xxxiii. 64; observations on its digestive functions, 353; observations on its sympathy with other organs, xxxiv. 66; observations on the utility of ipecacuanha in debility of the, xviii. 18; observations on inflammation of the, 67; reflections on its influence on the rest of the economy, 477.—*See* DIGESTION, GASTRITIS, *and* GASTRODYNIA.

STOKES, Dr. of Chesterfield, observations on the saccharine matter of the beet-root, iii. 8; remarks on the chemical doctrines of Mayow, 327; observations on erysipelas, and on its contagious nature, iv. 301; cautions respecting cow-pox inoculation, v. 17; observations respecting the nomenclature of cow-pox, vi. 386.

———, Dr. William, observations on pemphigus gangrenosa, xix. 344; observations on the effects of antimonial powder in cases of sanguineous determination to the head, xvii. 551.

STONE, Dr. A. D. review of his treatise on the disorders of the stomach, xv. 576; remarks on that work, xvi. 422; xviii. 18.

STONE, operation for the.—*See* LITHOTOMY.

STONES, in the urinary bladder.—*See* CALCULI.

———, *meteoric.*—*See* METEORS.

STORER, Dr. J. history of a case of small-pox after vaccination, xxvi. 378.

STRABISMUS, observations respecting its origin, xvii. 523, xix. 313.

STRAMONIUM, observations on its medical qualities, xxvi. 20, 48; xxv. 377; its beneficial qualities in the cure of asthma, xxvii. 22, 48, 150, 490.—*See* DATURA.

STRATTON, Dr. case of singular omental hernia, i. 156.

STRENGER, Mr. on the mode of administering galvanism in cases of deafness, ix. 290.

STRICTURE, of the ŒSOPHAGUS, case of, ix. 552; case of, relieved by tobacco glysters, xvii. 20.

———, *of the rectum*, cases of, with remarks, xxvii. 413; xxxi. 403; xxxiii. 236.

———, *of the urethra*, observations on, and on the use of the bougie in the treatment of it, i. 58; observations on the use of caustic bougies in the treatment of, ii. 303; on the advantages of that remedy, iii. 289; remarks on the use of bougies in the cure of, v. 514; remarks on the use of caustic bougies in, 573; on the particular efficacy of that remedy, ix. 190; cases thus cured, xiv. 474; a severe case of, xvi. 10; observations on the use of the caustic bougie in the cure of, xviii. 383; on the utility of tobacco bougies in the cure of, 351, 380; observations on their nature, origin, and treatment, xx. 314; on the efficacy of cantharides in the cure of, 429; further observations on their nature and treatment, xxi. 79, 161, 303, 371; xxii. 17, 126, 517; on the treatment of by the caustic bougie, xxi. 377; case of severe hæmorrhage from the urethra induced by the bougie, xxii. 341; comparative observations on the treatment of by the caustic and by the common bougie, 517; remarks on the treatment of by cantharides, xxiv. 313; remarkable case of, xxxi. 398; observations respecting the disposition to their recurrence, xxxii. 160.

STRINGHAM, Dr. description of a remarkable species of intestinal worm, x. 547; case of hydrocephalus, xiv. 42; case of extraordinary effects of mercury, xviii. 331; observations on digestion, xix. 420.

STROMEYER, Professor, account of his chemical tables, xix. 574.

STROWGER, Mr. reply to Harlestonensis, xx. 145.

STRUVE, Dr. his remedy for hooping-cough, i. 84; account of his translations and illustrations of Bacon's treatise on the prolongation of life, ii. 496; his essay on the art of resuscitation, ii. 497; account of his Triumph of Medicine, v. 175; review of his treatise on the physical education of children, 293; his treatise on asthenology, 377; his esssay on the art of restoring suspended animation, vi. 83; remarks on the extirpation of the uterus, xi. 34; account of his copy of the Angurapoy of Zoroaster, xiv. 91.

STUART, Dr. James, observations on the occasional injurious effects of leeches, xix. 57.

STUTZ, Dr. on the medical utility of alkalies, v. 470, 557; account of his mode of treating tetanus, xviii. 94.

STYPTICS, recipe for, for suppressing hæmorrhage, xvi. 191; a powder for the same purpose, xviii. 199; charcoal supposed to be a famous Flemish one, xxv. 497.

STYRAX, account of its medicinal qualities, i. 163.

SUBOFF, Mr. his mode of increasing the spirituous product in distillation, iii. 272.

SUE, M. his remarks on the punishment of the guillotine, i. 52.

SUERSON, M. on an easy mode of obtaining phosphoric acid, iv. 447.

SUFFOCATION, observations on, xiv. 150.—*See* Asphyxia.

SUGAR, mode of extracting it from the beet-root, ii. 188; iv. 249, 366; xvii. 197; from the stalks of Indian corn, 247; from the Siberian cow-parsnip, ibid; from the juice of the white and black birches, 248; from the musk of wine, ibid; from the turnip and carrot, 348; from the skirrit, ibid; from the parsnip, 349.

SUGURE, Mr. case of gastrodynia, iv. 227; observations on asthma, and on the agency of digitalis in pulmonary affections, iv. 329.

SULLY, Mr. observations on cow-pox, xvi. 23.

SULPHUR, case of dropsy cured by, i. 191; proposal for inhaling its vapours in cases of tubercles of the lungs, 192; on its efficacy in facilitating the combination of lard with mercury, ii. 389; test for the arsenic contained in, iii. 269; mode of preparing the white precipitate of, xiii. 171; account of a sulphurous spring, xxiv. 191; account of the preparation termed alcohol of, xxx. 79.

SULZER, his observations on galvanic influence of zinc and silver on the tongue before the discoveries of Galvani, vii. 70.

SUMACH, account of the poisonous influence of its effluvium on bees, xvii. 487.

SUNDERLAND, account of the diseases at in the autumn of 1803, x. 454.

SUNTER, Mr. S. on the application of cold in abdominal inflammation, xxx. 512.

SUPER-FŒTATION, an essay on, xvii. 189; observations on, with cases, xxii. 47, 293; xxiii. 278, 457; xxiv. 127, 232; xxxi. 150; observations on as relates to medical jurisprudence, xxxii. 334.

SUPERSTITION, reflection on its agency in favouring the influence of morbific agents, xv. 113, 117.

SUPPURATION, case in which thirty-six pints of pus were discharged from an abscess in the side, which terminated favourably, xii. 252; case of, of the liver to a great extent, terminating favourably, 497; case of extensive from the pluna, xviii. 393; observations respecting its existence without ulceration, xiii. 79, 321, 548.—*See* Abscess.

SURGERY, historical view of its progress in the sixteenth century, ii. 54, 155, 278, 364, 453; account of some particular cases in, v. 219; remarks on the operation of, v. 486; review of Mr. John Bell's Principles of, ix. 84, 177, 276; observations on select subjects in, ix. 197; review of a work termed Practical Observations on, x. 369, 466; xii. 176; observations on select subjects in, xiii. 7, 97, 289, 385; xiv. 481; xv. 1; xvi. 1; review of a collection of surgical essays, xvi. 271; observations on the principal operations in, xix. 481; theoretical suggestions for the improvement of, xxv. 146; observations on the state of amongst the ancients, xxxii. 111. 297; analysis of M. Boyer's work on, xxxiii. 298.

SUSPENSION by the neck, observations respecting the mode in which death is produced by it, xxxii. 469.

SUTLIFFE, Mr. observations respecting the efficacy of antimony and calomel in the cure of typhus, xxx. 321; observations on hooping-cough, xiv. 39.

SUTTON, Dr. observations on pulmonary consumption, iii. 560; his new theory of that disease, vi. 89; review of his work on remittent fever, xv. 189; remarks on his work on fever, xviii. 8; observations respecting the influence of temperature on consumption, xxiii. 202; observations on delirium tremens, xxx. 17, 404; observations on gout, 333; on the treatment of rheumatism, 362; on the Eau Medicinale d'Husson, xxxii. 178; on the use of elaterium and purgatives in the treatment of gout, 380; on the effects of temperature on consumption, xxxiv. 68, 89.

SWALLOW, (*hirundo*,) observations respecting the torpidity and transmigration of the, xix. 350; xxiv. 187, 256; xxv. 86.

SWALLOWING, difficulty of.—*See* Dysphagia.

SWARTZ, Professor, his arrangement of the mosses of Sweden, v. 370.

SWEAT, chemical analysis of the, xix. 124; enquiry concerning it, and on the agency of sudorifics, i. 287.

SWEATING-SICKNESS, researches respecting the nature of that once epidemic in England, xx. 473.

SWEDIAUR, Dr. review of his Pharmacopœia, xii. 565.

SWIFT, Mr. case of cutaneous eruption produced by taking internally Jesuit's Drops, v. 519; case of cardialgia cured by applying flannel to the region of the stomach, ibid; two cases of dropsy cured by digitalis, 348.

SWINE-POX, observations respecting it, iii. 219.

SYDENHAM, recommended in a forcible manner the utility of abstinence in the cure of acute diseases, i. 44; remarks on the excellence of his treatise on the small-pox, xiv. 196; remarks on his opinions respecting contagious and infectious diseases, xviii. 86; his opinions respecting the principal indication in the cure of the plague, xvii. 449; his remarks on the influence of the number of pustules in the excitement of fever in the small-pox, 572; his character as drawn by Cabanis, 567; remarks on his opinions respecting hydrocephalus and chorea, xviii. 220; his description of bronchitis, xx. 78; reference to some of his opinions respecting gout, xii. 150; xxvi. 190, 480.

SYER, Dr. remarks on the danger attending forcible delivery of the placenta, iv. 3; remarks on Mr. Simmons's case of monstrosity, v. 170; case of fracture of the cranium, 505; review of his treatise on the management of children, xx. 18.

SYLVESTER, Mr. observations on the Eau Medicinale d'Husson, xxiv. 351.

SYMES, Mr. observations on the influenza of 1803, x. 223.

SYMONDS, Dr. observations on the influenza of 1803, x. 224.

SYMPATHY, physiological and pathological observations on, xxiii. 244; xxxiv. 66.

SYMPHYTRUM, botanical description and medical qualities of, xii. 125.

SYNCOPE ANGINOSA, enquiry respecting its nature, iii. 389.—*See* ANGINA PECTORIS.

SYNDESMOLOGY, notice of an anatomical compendium thus termed, iv. 177.

SYPHILIS.—*See* VENEREAL DISEASES.

SYSIMBRIUM, botanical description and medical qualities of, xviii. 274.

### T.

TÆNIA, account of its destruction by electricity, i. 277; account of Matthieu's arcanum against the, v. 398; two cases cured by the use of sweet and bitter almonds, vi. 378; observations on, and account of a remedy for, ix. 290; a new mode of expelling the, xi. 434; the efficacy of oil of turpentine in destroying the, xxv. 167.—*See* WORMS.

TAINSH, Mr. cases of the plague occurring on board a British ship in the Levant, v. 538.

TAMUS, its botanical description and medical qualities, xiv. 68.

TANACETUM, its botanical description and medical qualities, xix. 353.

TANNER, Mr. observations on cow-pox, iv. 463.

TANNING, account of a mode of effecting it by pyroligneous acid, xxviii. 427.

TAR, account of its efficacy in the cure of tic douloureux, xxxii. 219; *water*, observations on its utility in venereal diseases, ix. 582.

TARANTULA, observations on the effects of the bite of the, xx. 224.

TARDY, Mr. observations on the treatment of insanity by Mr. Lucette, xxx. 124.

TAREINE, Mr. observations on the use of snails in hernia, xxiv. 18.

TARTAR-EMETIC, a convenient mode of preparing it, viii. 92.—*See* ANTIMONY.

TASTE, observations on its affection by galvanism, vii. 531.

TATHAM, Mr. remarks on the advantages of his truss for hernia, vii. 383.

TETTERS, observations on the efficacy of hemlock in the cure of, xi. 342.

TAUNTON, Mr. case of obliterated iliac artery, xxv. 35; observations on hydrophobia, xxi. 219; his mode of proceeding respecting vaccination, xxiv. 148; cases of disease of the testicle, xxxiii. 59; observations on the relative frequency of the occurrence of intestinal hernia at different periods of life, xxviii. 39; case of umbilical hernia, 13; case of puncture of the bladder terminating favourably, xxxii. 43.

TAXIS, for hernia, strictures on that operation, xx. 4.—*See* HERNIA.

TAXUS, its botanical description and medical qualities, xviii. 459.

TAYLOR, Mr. observations on the influenza of 1803, x. 225.

———, Mr. of Harleston, observations respecting the efficacy of cold water in the cure of gout, xi. 346; observations on the medical utility of digitalis, xx. 523; case of extraordinary abstinence, xx. 402; further observations on the use of digitalis, xxii. 124.

TAYLOR, Mr. of Wooton-under-Edge, his evidence in favour of vaccination before a Committee of the House of Commons, viii. 18.

TAYNTON, Mr. observations on the use of cold applications in gout, ix. 547; xiii. 141.

TEA, observations respecting its dietetic and medical qualities, xxiv. 189, 264.

TECHNOLOGY, medical remarks on, xxv. 56.

TEED, Mr. observations respecting the use of galvanism in the cure of rheumatism, viii. 552.

TEETH, remarks on the extraction of, with a description of some new instruments for, i. 13, 87; two cases of remarkable disease of the, iii. 55; remarks on the extraction of the vi. 251; review of a physiological essay on them in man and brute animals, viii. 558; observations respecting their vascularity, x. 360; remarks on the extraction of the, 240; cases of disease removed on extraction of diseased, 431; review of an essay on the natural history of the human, x. 77; observations on the extracting of the, and on filling cavities in, xviii. 210, 553; remarks on a natural history of the, xxxi. 302; remarks on the difficulties attending dentition in infants, iv. 75, 171, 267; v. 567.

——, pain of the, account of an efficacious popular remedy for, ii. 209; observations respecting another remedy for, ii. 209; observations respecting another remedy for, vi. 189; on the efficacy of pellitory of Spain in some cases of, v. 334; the tincture of the *coccinella semptem punctate* of Lin. recommended in, v. 546.

TEGART, Mr. history of a case of small-pox after vaccination, xxvi. 177.

TELLIGEN, Mr. observations on vaccination, xx. 334.

TEMPERAMENTS, reflections on the existence and nature of the, xiii. 30; xxi. 198.

TEMPERATURE, observations respecting the agency of diminished, on the animal economy, xiv. 267; observations on the relative influence of the different parts of the animal economy in regulating it, xxv. 180.—*See* Climate, Cold, *and* Heat.

TENERIUM, its botanical description and medicinal qualities, xvii. 466.

TERRAS, Mr. observations respecting his mode of treating venereal diseases, ix. 487.

TESTICLES, observations respecting the atrophy of the, prevalent in the army in Egypt, xii. 518; attributed to the heat of climate, fatigue, and the use of spirit distilled from dates, 519; remarks on those cases, xiii. 115; observations on cancer of the, 366; case of schirrus of the, xiv. 83, 529; cases of apparent want of development of the, xviii. 319; cases of fungous disease of the, with remarks, xx. 171; case of disease and extirpation of the, xxiii. 59; observations on some diseases of the, xxv. 175; observations on the symptomatic induration and enlargement of, 175; case of schirrus of the, 176; observations on sarcocele of the, 177; remarks on the effects of irritation of the urethra on, 265; case of sarcocele of the, 270; observations on schirrus of the, 273; observations on the sarcocele of Egypt, 289; observations on enlargement of, termed venereal, 273; observations on the scrofulous enlargement of, 274; observations on sarcocele of, 353; case of enlarged, from a blow, xxx. 165.

TETANUS, history of a case of, cured by the use of wine, iii. 569; observations on, and on the efficacy of opium in its cure, vi. 280; opiate friction advised in, vi. 479; case successfully treated by opium, vii. 55; history of a fatal case, from a wound, x. 345; case of, successfully treated, x. 492; case of, treated by opium, wine, cantharides, cold affusion, and mercury, termination fatal, chief morbid appearances on dissection, inflammation of the treachea, spinal marrow not examined, xiv. 129; case of, treated by cold affusion, termination favorable, 267; case of idiopathic, cured by the exhibition of opium, xvi. 473; case of, apparently arising from cold, treated by calomel, opium, oil of amber, mercurial friction, and wine, termination favourable, xvii. 211; observations respecting the good effects of opium and carbonate of potash in the treatment of, xviii. 93; case of, treated by cantharides and other stimulants, termination fatal, 443; observations respecting the utility of mercury in the treatment of, xix. 109; case of, from a wound, treated by cantharides, termination favorable, 423; various cases of, and observations on, 241; two cases, from wounds, treated by the warm-bath and mercury, termination fatal, xxi. 181; other fatal cases from wounds, 407, 410,

411; case of, treated by active purgatives, termination favourable, 418; case of, from a wound, in which mercury was unsuccessfully employed, 446; reflections on its nature, 447; case of, from a wound, in which amputation of the limb was performed, termination fatal, xxii. 120; case of, from a wound, for which the arm was amputated, termination favourable, 123; case of, from a wound, in which mercury was employed, termination fatal, 185; case of, from a wound, in which the tourniquet appeared to produce temporary alleviation, termination fatal, 324; severe case of, from a wound, treated by ether, opium, the warm-bath, wine, and mercury, termination favourable, 396; case of, from a wound, in which opium and calomel appeared to be productive of some benefit, 479; reflections respecting its nature, and on the cause of its fatality, 482; further observations and reflections on it, xxiii. 15; account of the appearances on dissection in a case of, 518; case of, from a wound, treated by tartar-emetic, calomel, opium, and mercurial friction, termination favourable, xxiv. 40; case of, successfully treated by ardent spirits and opium, xxv. 359; observations on the treatment of, xxvi. 482; case of, successfully treated by large quantities of opium and purgatives, xxviii. 154; case of, successfully treated by opium and the warm-bath, xxix. 100; observations on its analogy with hydrophobia, xxix. 272; case of, shewing the good effects of opium and ipecacuanha in, xxxi. 73; review of a treatise on the nature, causes, and treatment, of, xxxiii. 315; case of, successfully treated by copious blood-letting, xxxii. 164.

THACKERAY, Mr. history of a fatal case of poisoning by arsenic, iv. 292; case of spina bifida, iii. 104; case shewing the efficacy of cold water in the cure of some species of convulsions, x. 410; observations on cow-pox, xii. 231; further observations on the efficacy of cold water in the cure of convulsions, 508.

THALICTRUM, its botanical description and medicinal qualities, xvii. 372.

THELWALL, Mr. observations on the cause and treatment of impediment of speech, xiii. 450; observations on the expediency of dividing the frænum of the tongue, xiv. 256; further remarks on the management of impediment to speech, xv. 172.

THENARD, account of his mode of obtaining rape-seed oil, ix. 383; observations on the sebacic acid, ix. 78; memoir on vinous fermentation, xi. 411; chemical analysis of the perspirative fluid, and of milk, xix. 124; observations on muriatic ether, xx. 125; observations on the combination of acids with animal substances, xxiv. 321; observations on the power of the different galvanic piles, xxv. 362.

THERMOMETER, description of a new one, xiv. 572; proposal of a new scale for the, xxiv. 191.

THESAURUS MEDICAMINUM, critical notice of that work, vii. 564.

THIEBAUT, M. history of the blue disease, x. 575.

THIRST, physiological observations on, x. 20.

THLASPA, its botanical description and medicinal qualities, xviii. 174.

THOMANA, Dr. notice of his Annals of Clinical Medicine, ii. 484.

THOMAS, Mr. Charles, remarks on a case of diseased bladder, xvii. 34, 527.

———, Mr. H. Leigh, description of a hermaphrodite lamb, ii. 1; case of artificial dilatation of the female urethra, xxii. 155.

———, Dr. Robert, observations on the Gibraltar fever, xiv. 202.

———, Dr. T. observations on the heat evolved during inflammation, xxx. 322; analysis of some bitumen dug from the earth at Highgate, 343; account of his biography of Fourcroy, 499.

———, Mr. of Wakefield, remarks on the expediency of making pressure on the perinæum in cases of natural labour, iv. 441; on the good effects of ether and opium in cases of spasm of the stomach, 444.

———, Mr. of Pimlico, observations on vaccine inoculation, viii. 165.

———, Mr. W. observations on the Egyptian ophthalmia, xxi. 164.

THOMSON, Mr. Alexander, remarks on the insusceptibility to cow-pox, and small-pox, at particular periods, xiii. 51.

———, Dr. D. case of abscess of the liver, xxvii. 278; observations on inflammation of the liver, xxviii, 279.

———, Dr. Thomas, chemical analysis of sodalite, xxvi. 303; account of his history of the Royal Society of London, xxviii. 314; observations on the Botany-Bay gum, xxix. 474.

———, Mr. A. Todd, review of his Prospectus of the Pharmacopœias, xxiv. 80; review of his Elements of Pharmacy, xxvi. 321.

———, Mr. R. case of hydrophobia, xx. 396; observations on the case of Anne Moore, the reputed fasting woman, xxiv. 311.

THORESBY, Mr. observations on the influenza of 1803, x. 409.
THORNTON, Dr. R. J. his evidence in favour of cow-pox before a committee of the House of Commons, viii. 19; observations on cow-pox, 185; case of suspended animation by hanging, with remarks on the mode of treating such cases, xvi. 529; review of his Family Herbal, xxiii. 424.
THORP, Dr. observations on the influenza of 1803, x. 319.
THUNBERG, Professor, observations on the efficacy of the oil of cajeput in various diseases, i. 285.
THYNEUS, its botanical description and medicinal qualities, xvii. 566.
THYROID GLAND, physiological observations on it, xvi. 289.—*See* BRONCHOCELE.
TICA, Dr. C. remarks on the use of metallic preparations in the cure of intermittent fever, xi. 246.
TIBIA, account of a peculiar affection of, induced by fever, xxiv, 501.—*See* LEG, *and* BONE.
TIC DOULOUREUX, observations on its nature and origin, iii 575; review of Dr. C. Fothergill's treatise on, xiii. 367; case of, in which division of the affected nerves was inefficacious, xv. 230; cases of, cured by dividing the affected nerve, xvi. 178; case of, successfully treated, xvii. 388; on the good effects of volatile alkali in, 579; case of, cured by cicuta, xviii. 136; two cases of, successfully treated by the use of mercury, 176; case of, cured by calomel and opium, xx. 280; case of, successfully treated by the use of tar, xxii. 219; case of, cured by belladonna, 433; cases of, cured by arsenic, xxvi. 494; case of, cured by bleeding and arsenic, xxvii. 281; history and description of the, xxxi. 129, 380; cases of, cured by hyosciamus and oxyd of zinc, xxxi.
TEIKELL, Mr. observations on the influenza of 1803, x. 185.
TILESIUS, Professor, abridgment of the first part of his work on herpetic eruptions, xi. 230.
TILIA, its botanical description and medicinal qualities, xvii. 69.
TIMBREL, Mr. extracts from his Observations on the Management of Ruptures, x. 375.
TIME, metaphysical reflections on our perception of it, xx. 154.
TIN, observations on its chemical combinations, i. 197; observations on its medicinal utility, xxiii. 314.
TINEA CAPITIS, observations on the efficacy of the enula helenium in the cure of, i. 298; observations on its treatment, vii. 293; inveterate case of, viii. 554; history of a case of, x. 542; cases of, cured by compression by means of adhesive plaster, xiii. 8; observations on the treatment of, 9; history of the disease, 24; its character, 25; symptoms preceding it, 26; account of various modes of cure, 27; case of, which terminated fatally, from the use of a lotion of decoction of tobacco, xiv. 305; observations on it, 496; instance of its disappearance after vaccination, xviii. 346; case of, successfully treated, xxi. 177; reflections on, 328; observations on the utility of carbonic acid in, 385; observations on, xxii. 519, xxvi. 203.
TISSOT, Dr. notice of his being the first who treated properly on the medical application of electricity, i. 54; his treatment of persons apparently drowned, 62; account of his treatise on Dietetic Regimen in the cure of Diseases, 106.
T. M. observations on the treatment of burns and scalds, xvii. 1.
TOBACCO, account of its beneficial agency used as an enema, in a case of stricture of the œsophagus, xvii. 20; observations on its effects in some cases of difficult parturition, xviii. 337, 418; general observations on its effects on the human body, xxiv. 353; its history and uses, 445, xxv. 120, 319; remarks on the origin of the term, 479; observations on its good effects in psora, xxvii. 45; fatal effects of, used as a glyster, xxx. 339; fatal effects of, used as a lotion to the head, in a case of tinea capitis, xiv. 305; case of retention of urine relieved by it, xxxii. 526.—*See* NICOTIANUM.
TODE, Professor, account of the regimen directed by, in cases of hypochondriasis, i. 50.
TOMASSI, observations on the use of common salt in the cure of intestinal worms, xii. 283.
TOMLINSON, Mr. case of femoral aneurism, xviii. 508.
TONGUE, dissertation on the cause and nature of the furred appearance of it in some diseases, iv. 422; general observations on it, and on its diseases, v. 247; observations on morbid elongation of the, vi. 353, 435, 522; observations on its structure, and cases in which a portion of it has been removed by ligatures, xi. 269; case of a diseased portion of it removed by a ligature, xiii. 179; observations on dividing the frænum of it, in children, xiv. 256; case of remarkable protrusion of the, 271.—*See* RANULA.

TORMENTILLA, its botanical description and medical qualities, xvi. 26.

TOPOGRAPHY, *Medical*, observations on the utility of a general one of Great Britain, xvi. 51, 91.—*See* MEDICAL TOPOGRAPHY.

TORPEDO, account of the experiments of De Humboldt and Gay Lussac on the, xv. 295.

TORPIDITY, *of animals*, observations on, xix. 383; general observations on, xxiii. 502; observation on that of swallows, xxiv. 187.

TORRENCE, Mr. observations on scarlatina anginosa, xxiii. 413.

TOULURIN, Mr. observations on some small egg-like concretions in urine, xxx. 477.

TOURNIQUET, proposal for its application for restraining uterine hæmorrhage from the lower extremities, i. 23; description of a new one, for operations on the tongue, ix. 17; description of an improvement in its construction, xii. 336, 544; a proposed improvement in the construction of the, xiv. 492; remarks on a new one, xv. 149.

TOWSEY, Mr. observations on some peculiar effects of ipecacuanha, xxiii. 419.

TOXICOLOGY, report of the Institute of France on that of M. Orfila, xxxiii. 400, 471.—*See* POISONS.

TRACHEA, history of a wound of the, x. 5; case of division of the, viii. 162; two cases of foreign bodies lodged in the, xxiv. 269; observations on a disease of, somewhat similar to croup, xxxi. 358.—*See* CYNANCHE TRACHEALIS, *and* CROUP.

TRACTORS, (*Perkins's metallic*,) account of their use and supposed effects, iii. 277; iv. 100.

TRACY, Dr. observations, illustrated by two cases, respecting the co-existence of small-pox and measles, iii. 572; observations on tetanus, xvii. 211.

TRAGOPOGON, its botanical description and medicinal qualities, xix. 165.

TRAIL, Mr. observations respecting the utility of cold affusion in the treatment of the influenza of 1803, xiv. 226.

TRANCE, history of an extraordinary case of, xxvii. 227.—*See* CATALEPSY.

TRANERY, Mr. notice of his memoir on Electric Phenomena, viii. 477.

TRANSIERI, Dr. his memoir on difficulty of breathing, with remarks, intending to shew the influence of the moon on the human body, viii. 401.

TRAVERS, Mr. review of his Inquiry into the powers of Nature to repair injuries of the Intestines, xxvii. 55, 128; xxviii. 8; case of aneurism by anastomosis, xxviii. 57; observations on the cataract, xxxii. 65; observations on vision at different distances, xxxiii. 243; observations on cataract, xxxiii. 416.

TREMELLA, observations on the influence of wet and warmth on that plant, v. 397; its botanical description, xx. 272.

TREPHINE, description of a new one, viii. 482; another improvement in the, x. 239; observations on the use of the, i. 328; *see* CRANIUM, *and* FRACTURES: for observations on the use of, xxv. 487, *see* CRANIUM.

TRICHIASIS, observations on its treatment, x. 562.—*See* EYE.

TRIFOLIUM, its botanical description and medicinal qualities, xix. 78.

TRIPOLI, observations relative to the medical topography of, xxxi. 478.

TROMSDORFF, Professor, account of a ready mode of rendering alkalies pure, iii. 86; his analysis of the Siberian blue chalcedony, iv. 374; his mode of preparing muriate of barytes without kali, vi. 467, xi. 382; his mode of preparing ammoniated iron, vi. 408; his discovering of a new earth, 473; xi. 285; his mode of obtaining metallic cobalt perfectly pure, x. 95; chemical analysis of arseniated hydrogen gas, with its properties, xi. 422.

TROPICAL CLIMATES, observations on their influence on European constitutions, xxx. 226.—*See* CLIMATE.

TROTTER, Dr. observations on the means of destroying contagion, iii. 245; on nitrous fumigation, iii. 321; on vaccination, 524; on nitrous fumigation, 526; observations on the efficacy of citric acid in the cure of sea-scurvy, iv. 154; v. 74; observations on the use of cold affusion, 74; review of his Medicina Nautica, ix. 185, 282; review of his essay on Drunkenness, and its effects on the human body, xi. 568; case of gout treated by cold affusion, followed by vertigo, hemicrania, and dyspepsia; and another case, where fatal apoplexy ensued a few hours after the same application, xii. 237; observations, shewing the constitutional nature of gout, 238; case of gastrodynia, 289; further observations on that case, xiii. 132; remarks on the preceding case, 207; case of mollities ossium, 392; syllabus of his work on Indigestion and Nervous Diseases, xv. 213; observations on the medicinal qualities of ipecacuanha, xxiv. 60.

TRUSS, description of an improved one, xxxiii. 104 ; description of a new one for exomphalos ; report of the Truss Society of London, xxxiii. 244.

TRYE, Mr. Charles Brandon, review of his illustrations of some of the injuries to which the lower limbs are exposed, x. 88 ; observations on cow-pox inoculation, xii. 355 ; remarks on the comparative merits of small-pox and cow-pox, xv. 301 ; observations on lithotomy, xxvi. 72.

TUCKER, Mr. cases of extra-uterine pregnancy, xxix. 448.

TUBER, its botanical description and medicinal qualities, xxvi. 355.

TUDOR, Mr. observations on the influenza of 1803, x. 226.

TULLIDGE, Mr. case of disease of the liver, xxxi. 353.

TULIP-TREE, *(liriodendron tulipifera)*, recommended as a bitter tonic, iv. 376.

TULPIUS, extract from his case of premature menstruation, vii. 2.

TUMORS, observations on the treatment and removal of steatomous, i. 282 ; observations on a new method for their removal, 421 ; history of an extraordinary one within the abdomen, ii. 362 ; case of polypous tumor of the uterus, iii. 1 ; observations respecting a peculiar sort of, found in the head of new-born infants, iv. 446 ; case of encysted, situate on the eye-brow, v. 520 ; case of extraordinary, in the pudendum, vi. 309 ; case of schirrus of the breast, removed by resolution, vii. 49 ; case of ranula of the tongue, vii. 292 ; case of polypous tumors in the lungs, viii. 201 ; further observations on the same disease, 360 ; remarkable case of steatoma, situate in the perinæum, ix. 17 ; cases of semi-osseous, on the ribs, x. 162 ; observations respecting the formation of schirrous tumors, xii. 175 ; on the effects of various remedies on schirrus, 76 ; case of polypus in the antrum highmorianum, arising from a wound, xii. 5 ; account of a new classification of, 176, 266 ; remarks on the diagnosis of the various species of, especially of cancer, 411 ; on the various causes of their production, 414 ; case of semiosseous, situate on the ribs, 465 ; case of osseous, situate on the sacro-ischiatic ligament, xiii. 178 ; observations on steatomatous, 284 ; case of schirrus, on the testicle, 293 ; *see* TESTICLES : case of, in the tongue, 365 ; case of large adipose sarcomatous tumor, 388 ; case of steatomatous, situate in the breast, xiv. 82 ; case of polypus, 179 ; case of an osseous, in the orbit of the eye, 471 ; observations on polypus, xiv. 179 ; observations on schirrous, xv. 79 ; on steatomatous, 276 ; case of polypus in the nose, xvi. 7 ; case of remarkable tumor in the brain, 470 ; case of polypus in the nose, xvi. 7 ; case of tumors in the liver, xviii. 485 ; case of extraordinary, within the tunica vaginalis testis, 577 ; observations on glandular, xix. 363 ; case of polypus of the bronchial membrane, xx. 471 ; review of a treatise on, xx. 242 ; cases, with dissections, of various species, 483 ; reports of the effects of a peculiar regimen on cancerous and schirrous, xxi. 508 ; further observations on the same measures, xxii. 79 ; case of, indurated in the breast, 161 ; cases of schirrous, on the rectum, 281 ; case of cancer of the breast, 353 ; case of polypus of the heart, 511 ; observations on cancerous, xxiv. 72 ; case of steatomatous, in the cavity of the abdomen of a child, 105 ; of the clitoris, case of, xxv. 235 ; case of, situate in the tongue, 134 ; case of polypus in the vagina, xxvi. 38 ; case of, in the cavity of the chest, in a chicken, xxviii. 284 ; case of, encysted in the brain, 502 ; cases of glandular, in horses, removed by excision, xxix. 296, 391 ; observations on those of serous membranes, xxx. 73 ; of the uterus, 337 ; case of large one in the neck, removed with success, 353 ; observations respecting the diagnosis of those of the abdomen, xxxi. 78 ; cases of polypus of the uterus, xxxii. 340 ; case of fleshy tubercle of the uterus, 341 ; case of extraordinary, at the origin of the meatus urinarius, 427 ; case of fungous of the bladder and ureter, 156.—*See* CANCER, FUNGUS, FUNGUS HÆMATODES, *and* HYDATIDS.

TUNGSTEN, experiments and observations on its properties, i. 239 ; easy process for obtaining its acid, x. 479.

TUPPER, Mr. Martin, history of a remarkable disease of the heart, with some reflections on animal heat, viii. 497 ; galvanic experiments on warm and cold blooded animals, ix. 146, 235 ; on the sensations of light, produced by disposing metallic substances in various ways, 240.

——————, Mr. P. review of his essay on the probability of the existence of sensation in vegetables, xxvii. 417.

TURNBULL, Mr. case of hydrophobia, xix. 117.

TURNER, Dr. case of inflammation of the adipose membrane of the kidneys, xxi. 156.

TURNIP, observations on the mode of obtaining sugar from it, 4, 348.

TURPENTINE, *oil of*, observations respecting its efficacy in the removal of biliary calculi, i. 499; observations respecting its use in the cure of burns, iii. 206, 296; further observations, with cases, on the same subject, 262; iv. 454; v. 237; cases, showing its particular utility in the treatment of burns, xiii. 114; xiv. 34; 396; xv. 146; 458; xvi. 8; observations on the origin of this, as a remedy for burns, xvii. 131; history of a severe case thus treated, xviii. 304; another severe case thus successfully treated, 409; remarks on this mode of treatment, v. 55, 236; xv. 146, 342, 458; xvi 8, 115, 210, 460, 313; xvii. 1; xviii. 67, 159, 160, 238, 361; xix. 17, 229, 303, 417, 518; xx. 53, 54, 56, 491; xxi. 387; xxviii. 424; observations on its efficacy in the cure of intestinal worms, xxiii. 84, 128; xxv. 167, 546; xxviii. 81; xxx. 49, 129; observations on its successful employment in the treatment of yellow fever, xv. 16, 146; cases, with remarks, shewing its good effects in the cure of epilepsy, xxx. 509; observations, shewing its remarkable efficacy in the cure of puerperal fever, xxxii. 403; xxxiii. 179, 447; xxxiv. 183.

TURTON, Dr. observations on the influenza of the year 1803, x. 201; review of his Medical Glossary, xii. 563.

TUSSILAGO, its botanical description and medicinal qualities, xix. 359.

TUSSIS CONVULSIVA, observations on, and on the utility of stimulants in the treatment of it, xv. 222, 223.—*See* HOOPING-COUGH.

TUTROPA MULTIFIDA, observations respecting its utility in the cure of cancer, xxxii. 109.

TWINS (*fœtuses*), observations on the management of the delivery of, xxv. 29, 386, 480.—*See* DELIVERY.

TWITCH, Mr. observations on the swine-pox, iii. 219.

T. G. cases of severe cutaneous eruption somewhat like the psoriasis diffusa, xvii. 254; observations on the treatment of scalds, xviii. 67; on affectation in medicine, 253.

TYMON, Mr. cases of hydrophobia, xxviii. 351.

TYMPANITIS, case in which paracentesis was successfully performed in a child, vii. 223; Dr. Mead's description of this disease, vii. 232; historical account of the theories and treatment of this disease, and of the acupuncture mode employed by the Japanese in analogous affections, 225; severe case of, xiv. 267.

TYMPANUM, cases of successful puncture of the membrane of the, in deafness, xxii. 191; another successful case, xxxi. 346.

TYNICOLA, remarks on the nature and treatment of apoplexy, viii. 64; xi. 78; case of ischuria, xiv. 252.

TYROCINIUM MEDICAMINUM, review of the work thus termed, xxix. 494.

TYRO, observations on the nature and treatment of apoplexy, viii. 77; historical observations on the use of the ligature, xiv. 40; case of paralysis, xvii. 427.

### U.

ULCERS, observations respecting the efficacy of the balm of gilead in those affecting the viscera, &c. i. 34; observations on the treatment of inveterate, 183; suggestions respecting the means of destroying the virus of, 429; cases of inveterate, successfully treated by the use of angustura bark, i. 502; on the use of snails in fistulous, ii. 75; general observations on their cure, especially on the efficacy of the roller in their treatment, ii. 88; further observations on the same method, 195; observations on the particular efficacy of the plaster bandage in old ones of the legs, ii. 92, 197; case of obstinate, cured by friction with opium, 107; observations on a new mode of treating inveterate, 349; remarks on a particular species of, iv. 408; cases of malignant, successfully treated, v. 217; observations on the utility of the application of caustic alkali in foul and indolent, vi. 23; observations on inveterate, vi. 156; remarks on the use of the plaster bandage in the cure of, ix. 113; on the utility of the plaster bandage, x. 8; observations on the treatment of those of the cornea, x. 563; *see* EYE: observations on the phagedenic, especially relating to its comparison with chancre, 80; observations on a species of fungous, xiv. 514; remarks on those of the urethra, and on the question of their existence in gonorrhœa, xiii. 79, 321, 548; xvii. 573; remarks on the same subject, 321; observations on those affecting the glans, penis, and prepuce, 386; on the diagnosis of the various species of, 395; on the treatment of some resembling syphilis without mercury, 396; *see* VENEREAL DISEASES: observations on the good effects of cold applications in the cure of some species of, xx. 511; observations and remarks on the treatment of those of the legs particularly, xxiii. 432, 516; several cases of old ulcers of the legs cured by tonics and stimulants, xv. 217; observations on the use of house-leek in the cure of, xvi. 76; on the efficacy of mezereon in some species of, xiv. 544; further observations, shewing the utility of stimulants in the treatment of some species of, xxiii. 392; remarkable case of, in the groin and abdominal parietes, 221; cases of, consequent on venesection, 348; observations on those of the intestines, and remarks on their diagnosis, 386; observations on the treatment of those situate over the tibia, xvi. 77; remarks on their cicatrization, xvii. 572; remarks on the diagnosis of the various species, xviii. 82; on the utility of the nitrate of silver, in the treatment of some species of, i. 430; cases in which the nitrate of silver was successfully employed in the cure of, xix. 78; further remarks on their treatment by the nitrate of silver, xix. 97; observations on the use of the bandage in the cure of the, affecting seamen, xx. 110, 253; on the advantages of the paper bandage in some cases, xx. 256; observations on the use of cantharides in some species of, xx. 291; this application used by Galen, xxxii. 301; remarks on the influence of season on the state of, xxiv. 84; cases of severe, of the tongue, cured by nitric acid, 355; cases and remarks on the utility of nitre as a topical application in sphacelating, xi. 296; xii. 483; xiii. 324; xv. 97, 504; cases, with remarks, illustrative of the influence of disorder of the gastric system on the state of, xxix. 200; cases, with remarks, shewing the efficacy of carbonate of iron in the cure of some species of, xv. 477; xvii. 201, 325, 444; xviii. 16; xxii. 357; xxiv. 29; xxix. 13, 34, 332; on the utility of the juice of carrots in the treatment of some species of, xii. 232; particular account of the mode of using that remedy, xxiii. 140; xxxiii. 384; cases, shewing that ulcers of the intestines are some causes of chronic diarrhœa, xxxii. 374; observations on those situate about the origin of the toe-nails, xxxiii. 283, 462.—*See* WOUNDS, CANCER, *and* CUTANEOUS DISEASES.

ULEX, its botanical description and medicinal qualities, xix. 75.

ULENSIS, its botanical description and medicinal qualities, xii. 230.

UNIVERSITIES, general account of the German, xxvii. 385; remarks on the mode of obtaining medical degrees at some, xv. 295.—*See the respective towns, &c. where they are situated.*

UNGUENTUM NUTRITUM, account of the mode of preparing it, xi. 176.

UNZER, Dr. his opinions respecting the efficacy of emetics in persons who have swallowed narcotic poisons, i. 62; his treatment of erysipelas, 154.

UPAS ANTIAR, observations respecting its effects on the animal economy, xxvi. 252; xxxii. 343.

URANOLYTES, account of the fall of some near Agar, xxxiii. 215.—*See* METEORIC STONES.

URETERS, singular case of obstruction of the, i. 448.

URETHRA, observations and opinions respecting the existence of ulcers in, in gonorrhœa, xiii. 79, 321, 548; account of a singular disease of the, xiv. 469; case of dissection of the, after stricture, xvi. 107; objections to the opinion of the existence of ulceration of it in gonorrhœa, xvii. 573; case of severe injury of the, xxxix. 270; cases, with remarks, illustrative of the efficacy of cantharides in some diseases of the, xv. 415, 417; xvi. 78; xx. 429; case of severe laceration of the, xxi. 209; case of severe contusion of the, 367; observations on its contractile power, xxii. 17; case of extraordinary dilatation of it in a woman, xxii. 155; remarks on the opening into, from its external surface, for the extraction of calculi, &c. 24; description of two muscles surrounding it, 159; observations on the utility of cantharides in some diseases of the, xxiii. 311; case of congenital imperfection of the, 122; case of extraordinary dilatation of the, in a man, 524; account of a membranous septum situate at its extremity, xxv. 41; further remarks on that membrane, 48; observations on irritation of the, 265; case of rupture of the, from a contusion, xxviii. 245; remarkable case of stricture of the, xxxi. 398; on the disposition to recurrence of disease of producing stricture, xxxii. 100; observations respecting a thickening of the cellular membrane surrounding it in females,

341.—*See* Strictures, *and* Prostate Gland.

URINE, case of suppression of, relieved by the use of a decoction of the leaves of the peach-tree, iii. 563, case of, successfully treated by the application of blisters, vi. 526; case of incontinence of, x. 423; *see* Bladder, *and* Ischuria: account of some experiments on the composition of urinary calculi, iv. 467; further experiments on the same subject, vi. 527; chemical analysis of the constituent parts of the, vii. 474; chemical analysis of that of horses, viii. 476; experiments on that of patients with diabetes, xx. 12; further experiments on urinary calculi, 242; observations on, with remarks on the different analyses to which it has been subjected, 349; further observations on the composition of urinary calculi, 489, 499; chemical analysis of it in a case of diabetes mellitus, xi. 174, 338; chemical analysis of it, xix. 127; chemical, physiological, and medical, observations respecting it, xx. 548; chemical analysis of a urinary calculus found in a dog, xxi. 370; observations on the different species of calculi formed by the, xxii. 109; observations on its composition, and on the efficacy of magnesia taken internally in preventing the development of uric acid, xxiv. 203; observations respecting cystic oxide, xxv. 254; observations on vomiting of, xxxi. 19, 21, 26, 100, 125, 196, 205, 283, 359; observations on suppression of, 111; analysis of the urine in diabetes, xxviii. 63; analysis of that of the ostrich, 470; analysis of that of man in various diseases, 471; chemical analysis of it, xxx. 234; observations on the influence of the use of magnesia in altering its composition, 249, 324; analysis of a calculus found in the urethra of a hog, 256; observations respecting some small egg-like substances found in it, 477; observations respecting diabetic urine, xxxii. 435; analysis of the urine, in the state of health, xxxiii. 69; account of a peculiar kind voided by a man having gonorrhœa, 160; on the influence of alkalies, and the absorbent earths, on the properties of it, 445.—*See* Calculi, *Urinary*.

URINARY ORGANS, description of a congenital malformation of the, xiii. 179; attempt towards a systematic classification of those malformations in men, in which the ureters open externally on the surface of the abdomen, xiii. 179; case of malformation of the, 575; review of a treatise on the diseases of the, xxxi. 241.—*See the Distinct Organs, and* Monstrosities.

URTICA, its botanical description and medicinal qualities, xii. 31.

URTICARIA, observations on its nature and treatment, xx. 273.

UTERUS, historical observations on the anatomical discoveries respecting it, iii. 157; case of extraordinary action of it in the expulsion of a fœtus, 315; case of ossified, 422; anatomy of it in the gravid state, 478; case of extirpation of it, for inversion, iii. 554; case of inversion of the, successfully treated, v. 450; observations respecting sudden dilation of it during pregnancy, vi. 277; case of inversion of it, 503; account of a case of premature development of the, vii. 1; case of a violent affection of it, viii. 431; remarkable enlargement of it, 432; observations respecting the extirpation of it, and suggestions for the use of this measure in cancer of that organ, xi. 34; observations on a peculiar displacement of the, xi. 73; case of inverted, xi. 207; case of procedentia of the, xiii. 129; observations on the use of the sponge pessary in the treatment of procedentia of the, 130; xv. 21, 235; account of a case of rupture of it during parturition, which terminated fatally, 234; case of morbid enlargement of the, 387; case of prolapsus of it, xiv. 98; case of ossification of it, xv. 279; case of inversion of the, 385; observations on retroversion of the, xvi. 385; observations on wounds of it, 402; case of successful extirpation of it, xvi. 472; review of a treatise on the gravid, xviii. 22; observations on the treatment of hæmorrhage from the, xviii. 281; observations on the utility of blood-letting in cases of rigidity of it, during labour, xxiii. 337; case of mortification of it, from procedentia, xviii. 344; case of severe painful affection of the, xix. 550; case of recovery, after rupture of the, during labour, xix. 209; case of disease of, considered to be cancer, cured by cantharides, 528; case of remarkable disease of the, 551; case of inversion of the, with remarks on that affection, xxi. 64; case of remarkable disease of the, xxiii. 299; remarks on the degree of importance that should be attached to its functions in the state of health, 518; case of excision of it, terminating favourably, xxv. 210; observations on retroversion of it, and on extra-uterine gestation, xxiv. 406; observations on polypus of it, xxvii. 466; observations on the use of carbonate of iron in diseases of the, supposed to be cancerous, xix. 332; observations on the utility of opium in spasmodic affection of it, xx. 29; case of rupture of it during labour, 316; case of tumor in it, 337; general observations on the treatment of hæmorrhage from the, xxxii. 136; on the use of opium in that affection, 153; on the good effects of the secale cornutum in the same disorder, 97; cases of carcinoma of the, 240, 336; observations on procedentia of the, 241; on inversion of it, 244; remarks on the origin and treatment of the fleshy tubercle of the, 341; case of rupture of it during labour, terminating fatally, xxxii. 194; observations on polypus of the, 340; case of hydatids in the, xxxiii. 346; case of retroversion of it, 348; case of rupture of it, 359; observations on its state and appearance after impregnation, 335; case of remarkable disease of the, xxxiv. 102.

UTON, Mr. remarks on cutaneous diseases, xviii. 564.

UVA URSI, observations on its medicinal qualities, xxxi. 298; observations respecting its efficacy in the cure of pulmonary consumption, xiv. 461; xv. 346; xviii. 94.

UWINS, Dr. observations on venesection, iii. 227; observations on febrifuge medicines, and on their operation, iii. 51; remarks on the progress of medical knowledge, xii. 419.

### V.

VACCINATION.—*See* Cow-pox, *and* Inoculation.

VACCINIUM, botanical description and medicinal qualities of, xiv. 172.

VADE MECUM, *the Anatomist's*, notice of the fifth edition of that work, xi. 189; *the Physician's*, notice of, xxiii. 75.

VAGE, Dr. observations on the treatment of inveterate ulcers, ii. 349; observations and remarks on the treatment of syphilis, iii. 465; vii. 559; viii 172.

VAGINA, cases of imperforate, iii. 168; xii. 236; observations on procedentia of, xxxiii. 244.

VAIDY, M. historical researches on croup, xxxiii. 247.

VAJUCO DU GUACO, (an American plant,) its efficacy in rendering innoxious the poison of serpents, vii. 478.

VALENTINE, Dr. observations on the relative influence of different quantities of variolous matter introduced into the system, i. 280; remarks on the efficacy of hemlock in the cure of tetters, xi. 342; on the successful use of the same in a case of inveterate disease of the bladder, xi. 344; proposal of a reward to Dr. Jenner from the government of France, xviii. 341; observations on the use of the actual cautery, xix. 429; remarks on hydrophobia, xxii. 53; observations on the treatment of tetanus, xxvi. 482.

VALERIAN, observations on its efficacy in cases of spasm of the stomach, i. 50; its botanical description and medicinal qualities, xi. 445.

VALTELEN, Professor, notice of his system of universal pharmacology, iii. 576.

VANDIER, Mr. account of an analysis of the Kilburn water, vi. 240, 513; analysis of the composition of Smith's metallic bougies, vii. 238.

VAN MONS, Dr. account of his mode of preparing muriatic ether, vi. 16.

VAN SWIETEN, Baron, his historical researches respecting tympanitis, vii. 230.

VAPOUR-BATH, *the air-pump*, observations on its efficacy in the cure of several diseases, ix. 175, 484.—*See* Bath.

VAPOURS, *acid*, researches respecting their power to destroy contagious miasmata, x. 178.—*See* Acids.

VARGAS, Don Pedro d'Orbies y, account of his observations on the power of the juice of the plant *vajuco du guaco*, to render innoxious the poison of serpents, xvii. 478.

VARICELLA.—*See* Chicken-pox.

VARLEY, Mr. observations on some atmospheric phenomena, xvii. 485.

VARNISH, account of the mode of preparing it for anatomical purposes, xv. 391; xxii. 351.

VAUGHAN, Dr. John, of Wilmington, U. S. history of a remarkable case of madness, viii. 311; review of his essay on the fever which prevailed in the United States in 1802, x. 365; observations on the efficacy of olive oil as a purgative, xix. 51.

———, Dr. Walter, of Rochester, reflections on the danger of introducing unknown poisons into the human system, i. 449; reply to Mr. Pulley, ii. 405; history of an uncommon case of malformation, iii. 495; remarks on an obscure case of disease, viii. 230; observations on the use of arsenic in hydrophobia, xiv. 50.

VAUME, Dr. remarks on the advantages of small-pox over cow-pox, iv. 450.

VAUQUELIN, M. analysis of the honey-stone, vi. 189; observations on the mode of analysing minerals, 373; analysis of the earth eaten by the inhabitants of New Caledonia, viii. 191; analysis of a blue oxide of iron, 479; observations on the nature of the colouring matter of the blood, vi. 469; analysis of the cerumen of the ear, vi. 475; observations on the juice of the papaya (*caraca papaya*) employed in the Isle of Bourbon, against the tape-worm, ix. 79; analysis of the boracites, 92; experiments with gum kino, xi. 276; account of his discovery of phosphate of lime in several species of corn, ix. 584; account of his discovery of a new metallic substance blended with platina sand, xi. 479; analysis of belladonna, xxviii. 205; analysis of the urine, 470, 471; analysis of the substance of the brain, xxx 55.

VAZIE, Mr. description of his instrument for facilitating evaporation, x. 479.

VECTIS, observations on its structure and use, xxvi. 111.

VEGETABLES, account of an inquiry respecting the nature of the extractive principle of, iii. 172.—*See* PLANTS, *and* PHYTOLOGY.

VEINS, observations on the absorbent faculty of the, xxv. 45; on their muscularity, and on their appearance when afflicted with inflammation, xiv. 50; case of obliteration of one of the internal jugular, xxvii. 163; observations on those of the bones, iii. 551; review of Mr. Hodgson's work on the diseases of, xxxiv. 129.

———, *varicose*, case of, apparently arising from chronic inflammation, xvii. 297; observations on the consequences of the application of ligatures to, xxi. 422; observations on the mode of treating them by the application of a temporary ligature, xxvi. 503; observations on, of the bladder and urethra, xxxii. 155.

VEITCH, Dr. J. observations on vaccine inoculation, iv. 306; observations on fracture of the skull, with an extraordinary case, 513; observations on amputation at the hip-joint, xvii. 469; review of his treatise on ophthalmia, 479.

VENEREAL DISEASES, testimonies in favour of their treatment by nitric acid, i. 100; account of an ancient document relative to the, ii. 272; observations on the remedies employed in their cure, iii. 564; essays on them, and on their concomitant affections, 190, 275; criticisms on their treatment, 465; Dr. Rowley's remarks on their treatment, 480; case of syphilis successfully treated by nitric acid, iv. 183; review of a practical treatise on the, vi. 180; remarks on the treatment of, vii. 559; account of some researches respecting the efficacy of oxygen in the cure of syphilis, x. 90; observations on the treatment of venereal diseases, viii. 7, 172; review of an inquiry into some of the effects of venereal poisons on the human body, ix. 571; observations on the use of celandine (*chelidonium*) in the treatment of syphilis, xi. 139; remarks relative to the identity of the poison of syphilis and gonorrhœa, xiii. 78; observations on a disease resembling syphilis, xii. 263; observations on some of the exasperated symptoms of syphilis, 279; severe case of ulcerated bubo cured without the use of mercury, 280; case of phagedenic chancre exasperated by the use of mercury, ibid.; remarks on the removal of the symptoms of syphilis by the use of mercury before the perfect cure of the disease, 281; case of disease resembling syphilis, not relieved by mercury, but at length subsiding spontaneously, 504; query respecting the different appearance of the symptoms from various species of virus, 506; observations on the use of nitric acid in the cure of syphilis, xiii. 79; researches respecting the different diseases that have been confounded under this appellation, 79; sivvens and yaws considered as varieties of, ibid.; remarks on the characteristics of chancre, 80; queries respecting the natural history of syphilis; 89; observations respecting the period of their appearance in the American islands, ibid; remarks on the use of mercury in the treatment of, xiii. 395, 549; remarks on the uncertainty of the diagnosis of the primary ulcer attending them, xiii 395; several cases of, cured without the use of mercury; 396; observations on the use of hemlock in the cure of, xiv. 126; on that of the jacca or viola tricolor, for the same purpose, 431; observations respecting the diagnosis of syphilis, xv.

22; case of ulcer of the tibia from, xvi. 77; various observations and remarks relative to, 172; case termed pseudo-syphilis, xvii. 239; observations on the effects of various articles of the materia medica in the cure of, 375; observations respecting the diagnosis of, 476; observations on pseudo-syphilis, [illegible]; remarks on the various species of, 571; on the antiquity of their origin, 573; observations respecting the diseases confounded with syphilis, xviii. 82; observations on them, affecting pregnant women, children, and nurses, xx. 560; observations on the similarity of syphilis and leprosy, and on the origin of gonorrhœa and the purulent ophthalmy of infants, xxiii. 79; experiments tending to show the occasional identity of the poison causing chancre and gonorrhœa, xxii. 312, 391; account of a disease resembling syphilis, 432; observations on the use of opium in the treatment of syphilis, xxiv. 45; observations on the origin, nature, progress, and specific differences, of syphilis, xxv. 364; various observations on, xxvi. 28; notice of a treatise on syphilis, xxvii. 137; account of the existence of, at Otaheite, xxviii. 494; various observations on pseudo-syphilis, xxix. 417; review of a treatise on, xxxi. 408, 495; remarks on their character in Portugal, 496; review of a treatise on the specific distinctions of, xxxii. 229; history of a case resembling syphilis, 449; observations on diseases resembling syphilis, xxxiii. 193; remarks on the secondary symptoms of syphilis, 261; observations on the tubercular eruption of, 410; observations on the diseases which have been confounded with syphilis, xxxiv. 226, 454; observations on a remarkable case of, 265.

VENESECTION.—*See* Bleeding.

VENTENET, Mr. botanical account of the lime-tree *(tilia)*, v. 394.

VERAX, observations on the use of cold affusion in gout, xix. 535; reply to, by Dr. Kinglake, xx. 65; observations on the treatment of inflammation of the lungs, 242; case of contracted urethra, xxi. 367; observations on the cause of inflammatory diseases, xxiv. 230; observations on the treatment of chorea, xxv. 208.

VERBASCUM, botanical description and medicinal qualities of, xii. 130.

VERBENA, botanical description and medicinal qualities of, xvii. 468.

VERITAS, observations relative to medical reform, xv. 506; xvi. 349; reply to Harlestoniensis, xx. 132; H.'s reply, 266.

VERMICULAR DISEASE, affecting the skin, case of, xxiv. 162; affecting the intestinal canal.—*See* Worms.

VERREL, Mr. observations on cow-pox, xvii. 25.

VERTIGO, investigation respecting its causes, with an extraordinary case of, xi. 450; observations on its nature and origin, xix. 297.

VESICULÆ SEMINALES, case of induration of the, iv. 506.

VESSELS, observations on the umbilico-mesenteric, ix. 170.—*See* Arteries, Veins, *and* Absorbents.

VETCH, Dr. John, observations on the sensibility of the inflamed cornea to light, xx. 472; on the influence of the atmosphere in the production of ophthalmia, xix. 545.

VETERINARY MEDICINE, general observations on the management of cattle, i. 107; on the treatment of the rot in sheep, 108; review of a compendium of, viii. 466; review of a system of, xii. 278; observations on the diseases of the foot of the horse, xxiii. 338; xxvii. 425.

VIBORG, Professor, account of his experiments to determine whether brutes are affected with the small-pox, viii. 271.

VIBURNUM, its botanical description and medicinal qualities, xii. 558.

VICIA, its botanical description and medicinal qualities, xix. 78.

VIEUSSEUX, Dr. observations on paracentesis of the abdomen during pregnancy, vii. 40.

VIGORIENSIS, observations on vaccination, xv. 350.

VINEGAR, on the mode of using it in fumigation, i. 80; on the mode of concentrating it, ii. 109; on the preparation of the dulcified spirit of, iii. 267; new, of distilling it, iv. 411; new, of preparing concentrated, vi. 474; easy mode of preparing the acetic acid, vii. 476; a mode of concentrating it, xii. 14; observations on its use in various surgical cases, xiii. 497; *see* Burns: mode of preparing the aromatic vinegar, xiv. 573.—*See* Acids.

VIOLA, its botanical description and medicinal qualities, xii. 233; xxiii. 350; *tricolor*, recommended in the treatment of venereal diseases, xiv. 431.

VISCUM, its botanical description and medicinal qualities, xii. 32.

VIPER, history of the case of a person bitten by a, with remarks, vi. 481.

VIREY, M. observations on the different species of aliment, and on their influence on the human body, iii. 65, 153, 249; on the odours exhaled by living animals, vi. 339, 439.

VISION, case of singular illusion of, xiv. 107; remarks on distinct, at different distances, ii. 331; xxxiii. 243; remarks on nyctalopia, xxvi. 163; xxxiii. 63.—*See* Eye, *and* Optics.

VITALITY, reflections relative to the essential cause of, ii. 242, 321; on the properties of the vital power, ii. 127; researches respecting its origin, x. 253; observations and reflections relative to its origin, xxvii. 441; xxx. 58; some general remarks respecting, xxxi. 250; opinions respecting its origin, 431; exposition of Mr. Hunter's hypothesis of the cause of, xxxii. 419; researches respecting the origin of, and on the influence of the different organs of the animal body in its preservation, v. 476, 563; xxxii. 57; researches respecting the influence of the different nervous systems, especially in its preservation, xxvii. 375; xxxi. 417; xxxii. 56.—*See* Life.

VOICE, researches respecting that of birds, vi. 279; observations respecting the use of galvanism in cases of loss of the, viii. 256; on the use of moxa in the same affection, ix. 389; case of loss of, cured by purgatives, xxii. 252; observations on the efficacy of electricity in case of loss of, xxxi. 39; a remarkable case of loss of, xxxiv. 360.

VOISIER, Mr. observations on a displacement of the uterus, xi. 73.

VOIGHT, Mr. account of his mode of preparing acetic ether and dulcified spirit of vinegar, iii. 267.

VOLTA, Sig. description of his galvanic apparatus, iv. 113.—*See* Pile, *and* Galvanism.

VOLCANIC ERUPTION, account of, in the sea of the island St. Michael, xxviii. 28.

——— ISLAND, account of one near the Azores, xxviii. 80.

VOMITING, observations respecting the treatment of that of pregnant women, i. 284; remarks on the excitement of it in cases of apparent death, 59; case in which pins were thus ejected from the stomach, vii. 17; history of a singular case of, xxv. 308; observations respecting the possibility of urine being thus ejected from the stomach, xxx. 44, 176, 197, 355, 465, 481, 488; xxxi. 19, 21, 25, 106, 111, 114, 124, 196, 205, 283, 359; observations on the utility of mercury in some cases of, xxx. 100; remarks on the distinction between nausea and vomiting, 199; account of the experiments of M. Magendie on the mechanism of the act of, 207; xxxii. 429; observations and remarks on, by M. Maingault, 518, and by M. Marquais, 519; case of laceration of the stomach and duodenum from, xxxii. 64, 399; account of Mr. H[illegible]ter's observations on the act of, 400.—*See* Emetics.

### W.

WADD, Mr. S. observations on the efficacy of the cold affusion in the treatment of gout, ix. 205.

———, Mr. W. review of his work on stricture of the urethra, xxi. 79, 161.

WADLEY, Mr. observations on the influenza of 1803, ix. 515; observations on venesection, xiv. 306.

WAGSTAFFE, Mr. observations on the extraction of the placenta, iii. 421; observations on thoracic adhesions, 359; three cases of peculiarity in the cow-pox, 361, 532.

WARBLINGER, Mr. history of a case of rupture of the tendino-achillis, xiii. 494; observations on the effects of galvanism, xv. 151; case of dislocation of the patella, with a description of his peculiar bandage, iv. 285.

WAINWRIGHT, Mr. observations on the treatment of a case in which pins were swallowed into the stomach, i. 425; history of an uncommon tumor in the abdomen, ii. 362; observations on the use of belladonna in some diseases of the eyes, iv. 5; observations on cow-pox, xiv. 433.

WALCHEREN, report of the disease prevalent at, xxxiii. 183; observations on the fever at, 418; report of the fever prevalent at, xxv. 1.

WALDON, Dr. observations on the utility of carbonic acid gas in scarlatina, xvi. 552; observations on the nature and treatment of scarlatina, xxv. 35.

WALES, Mr. observations on vaccine inoculation, vii. 540.

WALKER, Mr. Alexander, his theory of phonics, hearing, &c. xix. 385; remarks on the principles of surgical operations, 481; sketch of a natural system of medical science, xx. 39; introductory lecture to his course of physiology, 99; his opinions respecting the use of the medulla of the bones, 286.

WALKER, Dr. Henry, observations on the ophthalmy prevalent in the army, xxv. 357.

———, Dr. John, letter respecting the remuneration of himself and Dr. Marshall for their services in the Mediterranean in promoting vaccine inoculation, ix. 62; remarks on Mr. Goldson's work on vaccination, xi. 506; xii. 44, 169; remarks on the causes of failure of vaccination, 45; remarks on Dr. Adams's observations on cow-pox, 332; on the appearance of various pocks after vaccination, 438; on the immediate efficacy of cow-pox in preventing small-pox, 467; on the spurious pustule from vaccination, 540; remarks on the report of some cases of failure of the prophylactic power of cow-pox, xiii. 53, 183; remarks on a case of malformation of the heart, 429; remarks on Mr. Dunning's opinions respecting cow-pox, 435; Mr. Dunning's reply, 342; reply to Eosaco, 539; physiological reflections on the cranium, with regard to its influence on the brain, xiv. 298; remarks on, by Mr. Pullev, 529; remarks on some opinions of Mr. Ring on cow-pox, x. 544; reflections respecting the functions of the spleen, xvii. 161; physiological and pathological reflections relative to the eye, 522; observations respecting our perception of time, xx. 154; on hydrophobia, 209; observations on the cellular structure of the vaccine vesicle, 337; some observations on vision, xxii. 307; reflections on some diseases of the thoracic viscera, xxiii. 22; observations on the rejected vaccination bills, xxxiv. 113, 367.

———, Dr. J. R. observations on the diagnosis of diseases from worms in children, xxiii. 290; observations on the utility of emetics in diabetes, xxxii. 446; case of peritonitis, xxxiv. 11.

———, Mr. Richard, observations on the utility of carrot poultices in the cure of ulcers, xxiii. 140; proposal for a new scale for the barometer, xxv. 52, 149; observations on pulmonary consumption, xxxi. 296, 366; remarks on the London Pharmacopœia of 1809, xxxii. 10; on cow-pox, 211, 289; remarks on the use of specific remedies, 293; observations on fractures and dislocations, 470, 473; on removing diseased tonsils, 474; on the operation of cupping, 476; observations on gout, xxxiii. 2; on pseudo syphilis, 193; on the treatment of wounds and ulcers, 383, 462; observations on the treatment of hæmorrhage, xxxiv. 21; on the analogy between small-pox and cow-pox, 295.

WALKER, Dr Sayer, review of his work on the constitution and diseases of women, ix. 279.

WALL, Dr. Martin, evidence respecting vaccination before a committee of the House of Commons, viii. 142; observations on the Malvern waters, xvii. 83.

WALLIS, Dr. remarks on his account of the origin and management of impediment of speech, xv. 172.

WALNUT-KERNEL, observations on the efficacy of its oil in the cure of herpatic and some other cutaneous diseases, ix. 94.

WALSH, Dr. account of a fever that prevailed in the garrison at Quebec in 1805, xv. 446; case of hœmatirrhœa, xxx. 339.

WALTER, Professor, review of his work on diseases of the kidneys and bladder, v. 264.

WANT, Mr. observations on the treatment of gout, xxvi. 188; observations respecting the composition of the *Eau Medicinale* for the cure of gout, xxxii. 77, 201, 393, 491; on the poisonous agency of the colchicum on cattle, 215; case of paroxysm of gout removed by the colchicum, 312; observations on the efficacy of cold air and drinks in the cure of catarrhal affections, 353.

WANTZEL, Dr. account of an orifice in the retina of the eye, v. 585.

WARD, Mr. of Manchester, observations on the use of opium applied by friction, i. 441; ii. 6; cases of vaccination, accompanied by some remarkable circumstances, 134; observations on the treatment of hernia, with cases, iv. 36; observations respecting the powerful effects of opiate frictions in allaying irritation, removing spasms, and procuring sleep, vi. 478; proposal for the use of opiate frictions in the treatment of tetanus and hydrophobia, 479; account of some researches to determine the mode of agency of opium, vii. 124; further observations and reflections on the same subject, 342, 357; the sedative quality of opium expressly considered, 497; remarks on its exhilarating qualities, viii. 325; remarks on the opinions of Brown respecting its mode of agency, 333; account of further experiments with opium on animals, 340; remarks on some theories of its agency, 389; further observations and reflections to prove its agency as an immediate sedative, ix. 335; reply to Mr. G. N. Hill, respecting the agency of opium, xi. 111; remarks on his opinions respecting the agency of opium, and its efficacy in the cure of tetanus and hydrophobia, xi. 538; further observations and reflections in support of his theory of the agency of opium, ix. 40; remarks on his theory of the agency of opium, 153; history of a case of elephantiasis, ix. 545; history of a case of cancer, x. 448; remarks on the strictures made on his theory of the agency of opium, xi. 111; further observations and reflections on the treatment of tetanus and hydrophobia by opiate frictions, 538; xvii. 353, 457; remarks on those observations, xviii. 258; review of his treatise on the efficacy of opiate frictions in spasmodic and febrile diseases, xxiii. 71; observations on the analogy between hydrophobia and tetanus, xi. 540; xxiii. 149.

WARD, Mr. of Woodchester, observations on the influenza of 1803, x. 521.

———, Mr. James, account of a woman living two years without food, xxx. 157.

WARDLEY, Mr. history of a case of hydrothorax, xxxii. 101.

WARDROP, Mr. history of a case of hernia, where the obturator artery surrounded the mouth of the sac, xvi. 81; observations on the utility of evacuating the aqueous humor of the eye in some cases of ophthalmy, xvii. 193; case of tumor within the tunica vaginalis testis, xviii. 577; review of his essay on the morbid anatomy of the eye, xx. 182; observations on the extraction of the cataract, xxi. 146; observations on the cure of palsy by titillation, xxviii. 224; case of tumor in the cavity of the thorax, with some observations on the diseases of serous and synovial membranes, xxx. 73; further observations on the evacuation of the aqueous humor of the eye, xxxi. 504; observations on some diseases of the toes and fingers, xxxiii. 151; case of preternatural joint treated by the seton, 507.

WARE, Mr. review of his treatise on purulent ophthalmia, xxiii. 77; observations on the staphyloma, and some other affections of the eye, xxv. 161; observations on muscæ volitantes, xxxiii. 411.

WARREN, Dr. J. C. history of a case of disease of the heart, xxiii. 265; case of hernia, 379; collection of observations on pathological anatomy, xxxi. 274; observations on the use of mercury in febrile diseases, xxxiv. 323.

WARREN, Dr. Pelham, observations on the use of opium in the treatment of diabetes, xxxi. 152.

————, Mr. S. history of a case of fungus cerebri, xvi. 149.

WARNER, Mr. observations on the treatment of hooping-cough, vii. 305.

WARNOCK, Mr. account of a case of sudden death, after a fall, xviii. 309; observations on the utility of adhesive straps in the cure of ulcers, xx. 110.

WAYTE, Mr. case of compound fracture of the arm, xxxi. 456.

WASHBOURN, Mr. history of a case of cow-pox suspended by varicella, ix. 370; history of a case of spina bifida, xxi. 65; case of tic douloureux, xxvii. 281.

WASHINGTON, General, account of his fatal illness and death, with remarks on the medical treatment, iii. 473, 490.

WASTELL, Mr. case of thoracic suppuration, ix. 427.

WASP, case of fatal effects from the sting of a, xiv. 479.

WASSA, Mr. observations on the influenza of 1803, x. 395.

WATER, observations relative to its physical constitution, xxviii. 433; review of a treatise on the properties of spring-water, x. 462, 570; observations respecting the effects of common water on the human body, and on the advantages of the use of distilled, xi. 462, 570; xii. 1; xv. 74; xxi. 508; xxii. 79; xxiv. 306; observations on the noxious effects of rain-water in particular seasons, xix. 494; beneficial effects of cold affusion in scarlatina anginosa, iii. 556; xi. 27; xix. 320; xxvi. 444; xxvii. 35; xxxiii. 358; on the utility of affusion with cold water in amaurosis and weakness of sight, iv. 179; historical sketch of the use of affusion with cold water, iv. 397; remarks on the external use of cold water, v. 12; utility of affusion with cold in the cure of some ulcers, vi. 19; its efficacy in the treatment of burns and scalds, iii. 191; v. 238; viii. 443; xvi. 17, 115, 210, 460, 461, 526; xvii. 1, 132, 220, 440; xviii. 67, 159, 160, 207, 238, 361; xix. 17; historical observations respecting the use of cold water in the treatment of burns and scalds, viii. 443; observations on the use of affusion with cold water in gout, vi. 457; several cases of its salutary application in the treatment of that disease, 458, 459; ix. 116, 125; further remarks on the advantages of that mode of treatment, 205; x. 255, 357, 377; objections to it, xi. 150, 154; additional testimony in favor of it, 346, 521, 523; xii. 63; objections to the practice, 237, 375; cautions respecting its use, 500, 502; opinions in favour of the practice, 563; successful use of, xiii. 141; remarks on the danger of that practice, 229, 231, 274, 355; case and observations in favour of it, 357; method of employing it, 360; defence of the practice, 437, 460, 512; xiv. 120, 192, 215; objections to it, 200, 455; remarks on that practice, 502; observations respecting the danger of that practice, xv. 181; case adduced to show its injurious effects, xviii. 53; remarks on that case, 163; observations on the advantages of that mode of treatment, xix. 535; observations relative to it, 536; xx. 65, 69; xxvi. 97, 369; defence of that practice, xxxiv. 6; objections to it, 184; reply to those objections, and remarks on the practice, 361, 366; historical observations relative to the use of cold affusion in the treatment of gout, v. 419; ix. 362; xi. 150; xii. 464; xviii. 165; observations respecting the use of cold water in the treatment of fever, i. 18; iii. 51, 304; iv. 26; remarks on the use of cold affusion in fever, v. 12; benefits of it in typhus fever, 75; reflections on its mode of agency, 578; Dr. Currie's observations on that mode of treatment, vi. 381; observations relative to it, vii. 39, 255, 341; viii. 51; remarks on the most efficacious mode of employing it, 160, 357; extraordinary success of that practice in the navy, ix. 9; cases of typhus, in which it was successfully employed, 282; benefits of the practice experienced in the Mediterranean, xi. 21; observations relative to the practice, used in the way of bathing, xiii. 564; xiv. 38, 85, 87, 90, 101, 179; xix. 15; xx. 85; xxii. 43, 230; observations in favour of cold affusion in fever, xiv. 203; in catarrhal fever, 226; benefits of the practice in typhus fever, xvi. 217; xviii. 197; xix. 271; remarkable history of its beneficial influence, xx. 151; exposition of that practice, by Dr. Jackson, 81; observations on the claims of Dr. Wright to the introduction of the practice, ibid.; on those of Dr. Currie, 82; account of its efficacy in Dr. Jackson's practice, 83; cases of its peculiar efficacy, 165, 167; improved method of employing it, 562; inefficacy of it in some cases, xxiii. 159; observations on its effects, xxvi. 398, 431; benefits of the practice at Jamaica, xxviii. 296; observations relative to it in general, xxxiii. 358; cases of fever cured by cold affusion, xxxiv. 175; observations on that practice in the cure of yellow fever especially, iii. 569; x. 40, 272; xv. 17; xix. 175; xxv. 360; xxviii. 296; historical observations relative to the use of cold water in the cure of fevers, i. 18; iv. 347; ix. 504; xx. 81; observations respecting the use of cold water in convulsive diseases, iv. 287; x. 410; xii. 508; xviii. 400; xxviii. 273; cases of its efficacy in the cure of those diseases, xv. 52, 497; in tetanus especially, iv. 287; xiv. 267; xv. 497; xviii. 261; observations respecting the efficacy of cold water in rheumatism, ix. 574; xi. 346; observations on the use of cold water in the treatment of ophthalmia, xii. 234; observations respecting the use of cold water in colic, xiii. 18; case of obstinate head-ache cured by cold water, xvi. 372; observations on the effect of cold on the body, and especially on the pulse, xvi. 367, 370; xvii. 93; xix. 112, 304; on the utility of cold water in the treatment of cancer, xvi. 383; observations on the use of cold water in hernia, xvii. 55; the use of cold water recommended in hydrophobia, xi. 459; xviii. 400; recommended in peritoneal inflammation, xxx. 19; in inflammation of the abdominal viscera, xxxii. 90; (*see* BATHS, *and* BATHING:) account of a process for preserving it good in long voyages, iii. 90; vii. 477; account of the Spanish vessels, the *alcazarras*, for purifying it, 174; observations respecting a mode of purifying it, vii. 477; on the purification of it by passing it through charcoal, xii. 96; another process for purifying it, xiv. 191; mode of filtering it, xxiii. 439.

WATERS, *mineral*, review of an essay on their medicinal efficacy, iii. 562; observations on the efficacy of those prepared artificially, i. 296, 363; account of an establishment at Paris for the preparation of artificial, with the analysis of those they are made to resemble, vi. 185; observations on the preparation of artificial, viii. 86, 488; review of a treatise on the chemical history and medical properties of several of the most celebrated, iv. 350; particular observations on those of *Aix*, iv. 458; of *Balaruc*, vi. 186; of *Bath*, review of a treatise on, x. 183; observations on their powerful effects, iv. 457; xii. 405; xv. 565; chemical observations respecting them, xvi. 283, 435; observation relative to those of *Bristol*, vii.

301; of *Carlsbad*, xvi. 504; of *Cheltenham*, mode of preparing artificial, x. 574; chemical analysis of, xx. 193; xxii. 15; observations on their medicinal qualities, xxiii. 508; of *Droitwich*, xxviii. 74; of *Dudley*, xxi. 439, 464; of *Durham*, xvii. 479; of *Flockborough*, xxv. 84; of *Geyser*, xxv. 362; xxxiv. 42; of *Harrowgate*, ii. 35; of *Kilburn*, vi. 240, 513; of *Lipetzk*, xvii. 486; of *Malvern*, xvii. 83; of *Melksham*, xxx. 299, 383; of *Moffat*, ii. 354; of *Pyrmont*, i. 50; of *Reading*, xii. 240; of *Sand-Rocks*, xxviii. 22; of *Spa*, xxviii. 17; of *Stow*, xxii. 345; on *sulphurous*, xiv. 191; xx. 136.

WATE, Dr. observations on hooping-cough, xxxi. 55.

WATER-FLAG, botanical description and medicinal qualities of, viii. 543.

WATERHOUSE, Dr. Benjamin, of New England, observations relative to the introduction of vaccination into that part of America, iv. 471; vi. 63; observations relative to the preservation of vaccine virus, vi. 327; observations on the merits of vaccination, viii. 543.

——————, Dr. of Boston, observations on cow-pox, iv. 563.

WATER-JAGS, a vulgar name for chicken-pox at Newcastle, xiii. 58.

WATER-SPOUTS, explanation of their origin given by Mayow, i. 298.

WATHERSTON, Mr. observations on the efficacy of concrete citric acid in the cure of scurvy, iv. 154.

WATT, Dr. review of his observations on diabetes, consumption, &c. xx. 547; remarks on his opinions respecting diabetes, xxii. 110; observations on the treatment of diabetes, 232; description of a new instrument for operating for the stone in the bladder, iii. 195; description of his machine for remedying distorted limbs, iv. 93; observations on the subject of distorted limbs, 498; v. 119, 426; observations on vaccination, v. 430; his claim to the invention of an improved aneurismal needle, xxv. 238.

WATKINS, Mr. T. observations on the efficacy of yeast in the cure of typhous fever, xvii. 203.

WATKINSON, Mr. two cases of inversion of the uterus, with remarks, vii. 433.

WATSON, Mr. observations respecting the utility of mercury in the cure of phthisis, xviii. 324.

WAWM, Mr. observations on the stimulant plan of treating burns and scalds, v. 54; history of a case of croup, xi. 227; observations on extracting, and on filling cavities, of teeth, xviii. 210.

WAX, account of a species from the Brazils, xxvii. 73.

WAYTE, Mr. observations on the necessity of accoucheurs, xxxiv. 450.

WEATHER, observations on the influence of varieties of, on disease, xiii. 310.—*See* ATMOSPHERE, *and* CLIMATE.

WEATHERHEAD, Dr. G. H. observations relative to the use of bleeding in suppression of urine, xxix. 271; observations on erysipelas, xxxi. 441.

WEAVER, Mr. case of apoplexia hydrocephalica, xv. 322; observations on injections for fistulous ulcers, 324; case of constipation of the bowels removed by external frictions, 325; case of headache cured by arsenic, 506; observations on apoplexia hydrocephalica, xvi. 335; observations on cancer and fungus hæmatodes, xviii. 534; observations on the use of arsenic in cutaneous diseases, xix. 206.

WEBB, Mr. observations on the treatment of ulcers of the legs, xxiii. 432.

WEBER, Mr. observations on the effects of opium on the human body, x. 435, 501.

WEBSTER, Mr. of New York, notice of history of pestilential diseases, i. 79; iii. 559.

——————, Mr. of Derby, remarks on the case of Anne Moore, the fasting woman of Tutbury, xxx. 108.

WEDEKIND, Professor, observations on death by the guillotine, i. 51.

WEIKHARD, Dr. observations on gout, and its connexion with hypochondriasis and hysteria, i. 151.

WELDON, Mr. history of a case of angina pectoris, xvi. 487.

WELLES, Mr. account of a new method of treating gout, xii. 180.

WELLS, Dr. W. C. historical account of the galvanic discovery, and of the publications which have appeared on that subject, vii. 70, 159; x. 53; history of a case of change of colour of the skin, xxix. 508; review of his essay on dew, xxiii. 232; observations on vision, xxvi. 247.

WELPER, Mr. case of inverted uterus successfully treated, v. 450.

WELSH, Dr. observations on the bodies of some persons poisoned by arsenic, xi. 192.

WENN, (bronchocele,) review of a treatise on the nature and origin of, v. 175.

WENDT, Professor, on the efficacy of the mesembryanthemum crystallinum in some diseases of the urinary organs, vi. 371; observations on the use of celandine in the cure of syphilis, xi. 139.

WENZEL, Professor, observations on the state of the brain in epilepsy, xxxiii. 382; on the treatment of fungus cerebri, 403.

WERNER, Dr. account of his apology for the Brunonian system, iv. 273.

WESTCOTE, Mr. observations on the proper period for amputation in different cases, xi. 68.

WESTENDORF, Dr. account of his mode of preparing the acetic æther and the dulcified spirit of vinegar, iii. 267.

WESTMEATH, Earl of, his letter to Dr. Jenner respecting vaccination, xiv. 256.

WESTON, Mr. observations on cow-pox, x. 514.

————, Mr. J. case of tinea capitis, xiv. 305.

WESTRUMB, Dr. one of the authors of what was called the improved phlogistic system of chemistry, i. 67; observations on sulphurous mineral waters, xx. 133.

WHALES, observations on the hearing faculty of, ii. 480.

WHALLEY, Mr. observations on vaccine inoculation, viii. 26.

WHATELY, Mr. Thomas, observations on the use of the bandage in the treatment of wounds and ulcers, ii. 88, 195; observations on fistula in ano, iii. 224; description of a new instrument for performing the operation for fistula in ano, 493; observations on the treatment of gonorrhœa virulenta in men, v. 379; remarks on Sir Everard Home's mode of treating strictures in the urethra, with his own plan of treating those diseases, 479, 572; review of his treatise on strictures of the urethra, xi. 475, 558; his opinion respecting the existence of ulcers in the urethra in gonorrhœa, xiii. 321; observations on luxation of the humerus, 521; review of his treatise on the nasal polypus, xiv. 139; review of his treatise on an affection of the tibia induced by fever, xxiv. 501; observations on the injection of hydroceles, xxvi. 110; observations on procedentia ani, xxvii. 269; review of his treatise on necrosis of the tibia, xxxiii. 510.

————, Mr. of Burton, observations on the influenza of 1803, x. 520.

WHITE, Mr. W. of Bath, observations on hydrocephalus, iii. 113, 325; observations respecting the efficacy of opiate friction in the cure of some disorders of the stomach and bowels, vi. 387; vii. 188; case demonstrative of the efficacy of that treatment, viii. 16; observations on a species of porrigo affecting the scalp, xxi. 132; observations on schirrus of the rectum, xxii. 281; case of cancer in the breast, 355; observations on the treatment of stricture of the rectum, xxvii. 413; observations on the use of arsenic in some cutaneous diseases, xxx. 296; on stricture of the rectum, 403.

WHITE, Mr. of Manchester, account of the gradation in man, and in different animals and vegetables, and from the former to the latter, i. 405.

————, Dr. A. case of precocity of the physical faculties in a girl, xxiii. 243.

————, Dr. J. E. United States, history of a case of disease of the brain, xiv. 247; cases of aneurism, xi. 396; xx. 264.

————, Mr. of York, the first proposer of the use of spirit of turpentine in the treatment of biliary calculi, stone and gravel, i. 499; observations on the treatment of gangrene, 501.

————, Mr. James, Veterinary Surgeon, notice of his compendium of the veterinary art, viii. 466; xii. 278.

WHITEHEAD, Dr. observations on the yellow fever, as it prevailed at Norfolk, United States, in 1801, x. 266.

WHITELY, Mr. observations on the influenza of 1803, x. 393.

WHITE-SWELLING, observations on the efficacy of galvanism in the cure of, viii. 257; ix. 135.—*See* SCROFULA, *and the different Joints.*

WHITFORD, Mr. description of his new spring truss, xxxiii. 104.

WHITLAM, Mr. observations on the best mode of preserving leeches, xii. 349; on the beneficial effects of arsenic in chronic rheumatism, xiii. 16; further observations on the management of leeches, 75; observations on the use of hemlock, 125; on epilepsy, 527; history of an instance of the deleterious effects of mushrooms, xv. 247.

WHITLOCK, Mr. observations on the influenza of 1803, x. 293.

WHITTLE, Mr. observations on the form of the couching needle, xv. 103.

WHITTE, Dr. history of a case of hydrocephalus, xv. 28.

WHYTE, Dr. Douglass, observations on the treatment of inflammation of the eyes, vii. 209; on the prevention and cure of dysentery, 283; iii. 231; observations on cow-pox as practised at Constantinople, v. 243; observations respecting the plague, v. 243; xxvii. 271.

————, Mr. W. A. observations on the nature, causes, prevention, and cure, of gout and rheumatism, v. 288.

WICHMANN, Dr. observations on the febris urticaria, x. 481; his theory of dentition in children, iv. 171, 267; observations on dysphagia, ix. 293.

WIEDEMANN, Prof. notice of his Archives of zoology and zootomy, vi. 79.

WIEGLEB, Prof. one of the founders of the new system of chemistry advanced in Germany, i. 67.

WIESENTHAL, Dr. observations on worms found in poultry, ii. 204.

WIGARD, Dr. observations on the efficacy of calomel and musk in the cure of croup, xxxii. 80.

WILDENOW, Prof. historical account of the Balm of Gilead, i. 33; observations on the aromatic wood of the aloe, with a botanical description of the excoecaria agallocha, and the aquilaria ovata, ii. 293; observations respecting the plants from which the caoutchouc is obtained, xi. 398.

WILKINSON, Mr. G. of Sunderland, observations respecting the use of the air-bladder of fishes, ii. 360; remarks on the treatment of fractures, vi. 397; remarks on a case of apoplexy, related by Dr. Langslow, vii. 138; reply, 275; further remarks on that case, 436; observations on the influenza of 1803, x. 399; notice of his observations and experiments on the medicinal qualities of the bark of the broad leaved willow, 374; account of the diseases prevalent at Sunderland in the autumn of 1803, 454; further observations on the willow-bark, xi. 400; xii. 373; observations on the preservation of leeches, 485; remarks on Mr. Cumming's reply to Dr. Scott respecting the use of sulphat of zinc in ague, xiii. 135; reply of Mr. Cumming, 325; observations on the use of sal ammoniac and vinegar in various chirurgical diseases, 497; reply to Mr. Cumming, xiv. 46.

——————, Mr. C. H. observations on the medical powers of electricity, ii. 9; review of his Elements of Galvanism, xiii. 82, 371.

WILL, observations respecting its power over the act of respiration, vi. 372.—*See* Muscular Motion.

WILLAN, Dr. observations on the diseases of London, iii. 297; review of his reports on the diseases of London, v. 482; observations on cutaneous diseases, vi. 297; further observations on cutaneous diseases, xii. 97; review of part of his work on cutaneous diseases, xiv. 362; review of his treatise on vaccine inoculation, xvi. 372; review of the second part of his work on cutaneous diseases, xx. 273, 371; biography of, xxviii. 477; remarks on some of his opinions on cutaneous diseases, xxx. 413; review of his treatise on porrigo and impetigo, xxxiii. 128.

WILLICH, Dr. observations on the treatment of inveterate ulcers, i. 502; account of his lectures on regimen and diet, ii. 301, 393, 493.

WILLIAMS, Mr. A. account of the successful treatment of a case of suspended animation, iii. 223.

——————, Mr. of Aberystwith, observations on the influenza of 1803, x. 294.

——————, Mr. of Bridge-end, observations on the influenza of 1803, x. 230.

——————, Mr. of Merthyn, observations on the influenza of 1803, x. 289.

——————, Mr. R. description of a steam apparatus, xii. 516; remarks on the use of friction with oil in yellow fever, xiii. 18.

——————, Mr. J. T. remarks on Dr. Roberton's opinions respecting stricture of the urethra, xxiv. 313; observations on the functions of the liver, xxvii. 508.

WILLEY, Dr. observations on hooping-cough, xvii. 293.

WILLOW, botanical description and medicinal qualities of the different species of the, xi. 442; observations and experiments respecting the medicinal qualities of the bark of the broad-leaved, i. 190; x. 374; xii. 373.

WILMER, Dr. observations on luxation of the bones, xxiv. 468; history of a case of periodical catalepsy, xxviii. 169.

WILSON, Mr. James, account of two muscles surrounding the urethra, xxii. 159, 333.

——————, Dr. A. Phillips.—*See* Philip.

——————, Dr. D. of Richmond, U. S. history of a case of intermittent fever cured by calomel, xix. 15.

——————, Mr. John, observations on venereal diseases, xvi. 172; remarks on the diseases and depopulation of Otaheite, 174; on the secondary symptoms of syphilis, xxxiii. 261.

——————, Dr. of Montrose, observations on vaccine inoculation, iv. 340.

——————, Sir Robert, observations on the plague, xii. 91.

——————, Mr. J. of Gillingham, history of a case of lumbar abscess, viii. 44; further observations on that case, 229; letter to, from Dr. Vaughan, of

Rochester, 230; remarks on the price of medical books, x. 334.

WINCH, Mr. account of his mode of dissolving elastic gum, ii. 83.

WIND, in the intestinal canal, pathological dissertation on, iv. 556.

——, of a ball, observations on the supposed accidents from the, xxvii. 323; xxviii. 142; xxix. 63; xxx. 246.

WINDS, observations on the influence of them on contagion, xxxiv. 409. —*See their specific appellations, and* ATMOSPHERE.

WINE, observations on the means of detecting adulterations of, iii. 171; observations on its efficacy in the treatment of tetanus, 569; in the treatment of fever.—*See* FEVER, SCARLATINA, &c.

WINDSOR, Mr. history of a case of discoloration of the cornea, v. 241.

WINTERBOTTOM, Dr. T. M. observations on vaccination, vi. 46; vii. 294; remarks on the treatment of a case of apoplexy, xi. 78; review of his medical directions for the use of navigators and settlers in hot climates, x. 83; observations on cow-pox, xiv. 22; history of a case of pemphigus, xviii. 43.

WISHART, Mr. history of a case of disease of the heart, xxiii. 242; history of a case of fungus hæmatodes, xxv. 493; observations on the mode of operating for the cataract, xxx. 68.

WITT, de, Dr. account of some extraordinary effects of the stramonium, i. 84.

WITTER, Mr. observations on the beneficial agency of oxygen gas in restoring suspended animation, xxxii. 36.

WITMANN, Dr. notice of his Travels in Turkey and Egypt, viii. 285.

WOLCOT, Dr. his project of a treatise on the general causes of deafness, iv. 187.

WOLLASTON, Dr. account of his discovery of rhodium, xiii. 98; description of his blow-pipe, xvii. 93; experiments on the influence of electricity in inducing animal secretions, xxii. 174; account of his Croonian lecture in 1810, xxiv. 485; observations on cystic oxide, xxv. 254; experiments on the urine of persons having diabetes, 277; experiments on the blood of diabetic patients, xxvi. 462; observations on chemical equivalents, xxxi. 107.

WOMEN, review of a work on the constitution and diseases of, ix. 279.—*See* FEMALES.

WOOD, Dr. J. of Newcastle, observations on vaccination, xiii. 57, 61; observations on the use of muriate of lime in the cure of scrofula, xiv. 80; observations on cow-pox, xv. 135; history of a case of confluent small-pox, 532; observations on confluent small-pox, xvi. 62; observations on the treatment of burns and scalds, xix. 232; on the seasons attending epidemic diseases, 317; remarks on his observations respecting burns and scalds, by Dr. Kentish, 417; reply, 518; observations on hydrophobia, xxi. 134.

WOOD, Mr. Kinder, history of a case of injury of the urethra, xix. 217.

——, Mr. of Manchester, history of a case of Cæsarean operation, with the appearances on dissection, vi. 346; history of a peculiar kind of painful subcutaneous tubercle, xxviii. 506; history of a case in which a foreign body was found in the heart, xxxii. 37.

——, observations on the illumination of rotten, iv. 83; v. 583.

WOODCOCK, Mr. observations on the treatment of phymosis, iii. 162.

WOODFORD, Dr. of Castle Cary, case of small-pox after cow-pox, v. 151.

WOODFORDE, Dr. of Ansford, history of a case of apoplexia hydrocephalica, viii. 62; observations on the influenza of 1803, ix. 505.

WOODHAM, Mr. remarks on M. Larrey's cases of atrophy of the testicles, Egyptian ophthalmy, &c. xiii. 150; observations on the treatment of ganglia by escharotics, xxiii. 432; remarks on the discrepancy of medical opinions, xxxii. 183; on the folly and danger of some medical hypotheses, 457.

WOODVILLE, Dr. his reports on vaccine inoculation, i. 323; observations on the cow-pox, iv. 254; his efforts to introduce vaccination into France, iv. 470; observations on vaccine inoculation, v. 6; his testimony before a Committee of the House of Commons in favour of vaccination, vii. 491; funeral oration on him, xiv. 85.

WOODWARD, Mr. observations on infantile diseases, iv. 41.

WORMS, *intestinal*, observations respecting the influence of electricity in destroying them, i. 277; observations respecting the character of, ii. 149; account of some found in poultry, 204; observations on the efficacy of stizolobium (cowhage) in destroying them, iv. 267; account of the Prussian remedy for, v. 398; observations respecting the use of aloes and lime-water against ascarides, vii. 257; observations respecting the juice of the papaya, employed as a remedy for tænia in the Isle of Bourbon, ix. 79; description of a remarkable species, x. 547; account of a new mode of expelling them, xi. 434; remarkable instance of presence of ascarides in the biliary duct of a child, xv. 28; account of the Indian remedy for the tænia, xvii. 189; case in which an extraordinary quantity of ascarides was voided, xx. 380; observations respecting the efficacy of cannabina, (*agrimonia*, Lin.) against the tænia, xxiv. 517; observations on the effects of camphor on the tænia, xxv. 23; on the efficacy of spirit of turpentine against, 546; cases of, shewing the efficacy of oil of turpentine against, xxviii. 91, (*see* Turpentine); instance of the simultaneous existence in the intestines of eight tape-worms, xxxiii. 7; description of the ascarides present in the intestines, xxvii. 512; observations on the anatomy and physiology of the *lumbrici*, xxxii. 347; cases of the tænia in infants being destroyed by the decoction of pomegranates, 395; instances of their existence in the vagina and in the ear, xviii. 379; observations on the origin of, xxvi. 273; observations in favour of the equivocal generation of, 454.

WOUNDS, observations on the treatment of, ii. 88; remarks on the nature and consequence of those of the uterus, 232; historical observations relating to the treatment of, 55; particular remarks on the treatment of, 195; further remarks relating to wounds of the uterus, 438; historical observations on the treatment of, 278, 364, 365, 479; observations on the inconvenience attendant on the healing of, by the first intention, iii. 12; case of a remarkable occurrence in the stump, after amputation, 4; observations respecting the utility of gastric juice as an application to, iv. 167; observations respecting those of the clitoris, v. 1; case of, of the larynx, 346; observations respecting those of the œsophagus, vi. 97; on those of the uterus, 346; case of, affecting the trachea, viii. 162; observations on the adhesive process in, ix. 85; observations relative to those affecting the trachea, xiv. 516; observations respecting those affecting the abdomen and thorax, x. 250; case of, of the omentum, 356; observations respecting those affecting the bladder, xi. 3, (*see* Lithotomy); case of extensive, at the base of the skull, xiii. 270; case of, affecting the lungs, xviii. 323; case of death from those inflicted by military punishment, 176; cases of severe, of the lungs, 333; observations on the adhesive process in, xix. 563; case of lacerated, of the urethra, xxi. 209, 485; case of, of the femoral artery, 317; case of severe, of the abdomen, 414; case of severe, penetrating the cavity of the chest, xxvii. 464; observations on the influence of disease of the gastric system on, xxviii. 200; case of severe, penetrating the cavity of the chest, xxix. 68; observations on those of the intestines, xxviii. 8; case of, affecting the heart, 59; observations on those occurring in seamen, 87; case of, affecting the urethra, 245; observations on the process of healing of, xxxiii. 356; observations on the advantages of exposing them to the air, xxxi. 450; various observations on the treatment of, xxxiii. 383, 462; xxxiv. 21.—*See* Gun-shot Wounds, Ulcers, *the appellations of the various operations, and the diseases for which they are performed.*

WRIGHT, Dr. William, observations on the use of cold affusion in the treatment of small-pox, xix. 272; his claims to the original use of cold affusion in typhous fever, xx. 81.

———, Mr. observations on medical reform, xxx. 452.

WRISBERG, Dr. notice of his medical, physiological, anatomical, and obstetric, commentaries, vi. 77.

WRIST, case of contraction of the, with remarks, xxxii. 146.

WURTZBURG, notice of the annals of the clinical institution at, ii. 484.

WURZER, Professor, account of his process for purifying potash, i. 300; account of his treatment of syphilis by the nitric acid, iv. 183.

WYER, Dr. observations on phlegmasia dolens, puerperarum, xxiii. 301.

WYNNE, Mr. case of hydrophobia cured by bleeding, xxix. 285.

### X.

XANTHIUM, its botanical description and medicinal qualities, xii. 331.

XYLOBALSAMUM, observations on the, i. 34.

### Y.

YAWS, observations on the origin and nature of, xiii. 19; observations on, by Dr. Adams, xv. 382; further observations on, xx. 401; account of the mode of cure used by the negroes, xix. 122.

YEAST, observations on the medicinal qualities of, ii. 374; iii. 105; on the efficacy of, in the cure of typhous fever, iv. 539; v. 143; observations on its efficacy in malignant fevers, with tests to demonstrate that its basis is carbonic acid gas, 422; efficacy of, in a case of putrid fever, 508; observations on its efficacy in typhous and putrid fevers, vi. 1, 2; xvii. 503; account of a method of producing it originally, xx. 404.

YEATMAN, Mr. J. C. account of a case of recovery from the effects of a large dose of opium, xxxi. 468; remarks on the bad effects of bleeding in cases of poisoning by opium, xxxii. 371; history of a case of aneurism cured by pressure, bleeding, and digitalis, xxxiii. 377; account of a case of injury of the hip confounded with dislocation and fracture, 469; remarks on the state of medicine in Sicily, xxxiv. 186, 353; account of the sirocco wind, 355.

YEATS, Dr. observations respecting the use of abstinence from liquids in some diseases of the lungs, and the use of that measure by Dr. Lower, i. 20; remarks on the chemical discoveries of Mayow, 326; reply to Dr. Lubbock respecting the chemical discoveries of Mayow, ii. 99; observations on the use of nitrous fumigation, iii. 429; on the good effects of resuscitated salivation, xxx. 37; observations on ischuria and on vomiting of urine, xxix. 353; xxx. 44, 488; xxxi. 26, 106; history of a case of hydrocephalus, xxxiv. 198.

YELLOLY, Dr. account of a peculiarity in the course of the radial artery, v. 283; history of a case of œdema fugax, vi. 168; account of a tumor in the brain, xxii. 161; history of a case of death from arsenic, 427; history of a case of anathesia, xxx. 167; observations on a vascular appearance of the stomach after death, sometimes mistaken for inflammation, xxxii. 155.

YEO, Mr. observations on the influenza of 1803, x. 224.

YONGE, Mr. observations on the influenza of 1803, x. 221.

YORK, account of the hospital at, viii. 123.

YOUATT, Dr. observations on indigestion, xxxi. 231.

YOUNG, Dr. reply to the Edinburgh reviewers, xxxi. 12.

———, Mr. W. C. case of fœtus found in the abdomen of a boy, xxii. 166.

———, Mr. Samuel, review of his enquiry into the nature and action of cancer, xiv. 465; account of a canulated catheter, 490; his letter to Dr. Adams on cancer, xv. 393; his letter to Sir Everard Home respecting a case related in his work on cancer, 500; reply to Dr. Adams's support of his doctrine of cancer, xvi. 403; review of his treatise on the adhesion of wounds, xix. 563; review of his work on cancer, xxxiv. 410.

YOUNG, Mr. Thomas, his translation of Mr. Manuori's memoir on the organization of the iris, xvii. 403.

YOUNGLOVE, Mr. on the analogy of the motion of the blood in the arteries, and water in troughs and conduits, x. 284; remarks on the small-pox and cow-pox, xi. 317.

### Z.

ZADIG, Dr. history of the cure of ambliophia in a man sixty years of age, by the use of galvanism, x. 58.

ZANTHROXYLON, account of its efficacy in the cure of ulcers, ii. 30; its efficacy in colica pictonum, 33; further account of its efficacy in the cure of ulcers, viii. 453.

ZEOLITE, observations on, xxv. 361.

ZEZEGERY, or Sesasum, account of its use in the West Indies, in cases of dysentery, viii. 553.

ZINC, *sulphate of*, observations on its efficacy, combined with opium, in the cure of dysentery, ix. 55; observations on its efficacy in the cure of intermittent fevers, x. 476; its use, externally, in cutaneous ulceration, xi. 93; further observations on its efficacy in the cure of intermittent fevers, 248; remarks on its use in those disorders, xiii. 138, 326.

ZOONIC ACID, observations on, by M. Berthollet, i. 85.

ZOONOMIA, defence of the doctrines of the, ii. 276; remarks on the doctrines of the, iii. 139.—*See* Darwin, Dr. Erasmus.

ZOROASTER, some account of his Angurapory, a work thus termed, attributed to him, xiv. 91.

ZOSTERA, its botanical description and medicinal qualities, xvii. 71.

ZURINE, M. account of his observations respecting the water-flea, (monoculus pulex,) vii. 191.

